# Supplementary material for: Chemoenzymatic indican for light-driven denim dyeing
Source: Nat Commun. 2024 Feb 27;15:1489. doi: 10.1038/s41467-024-45749-3 (PMC10899603; doi:10.1038/s41467-024-45749-3)
Supplement: Supplementary file 1 — Supplementary Information [file 41467_2024_45749_MOESM1_ESM.pdf]

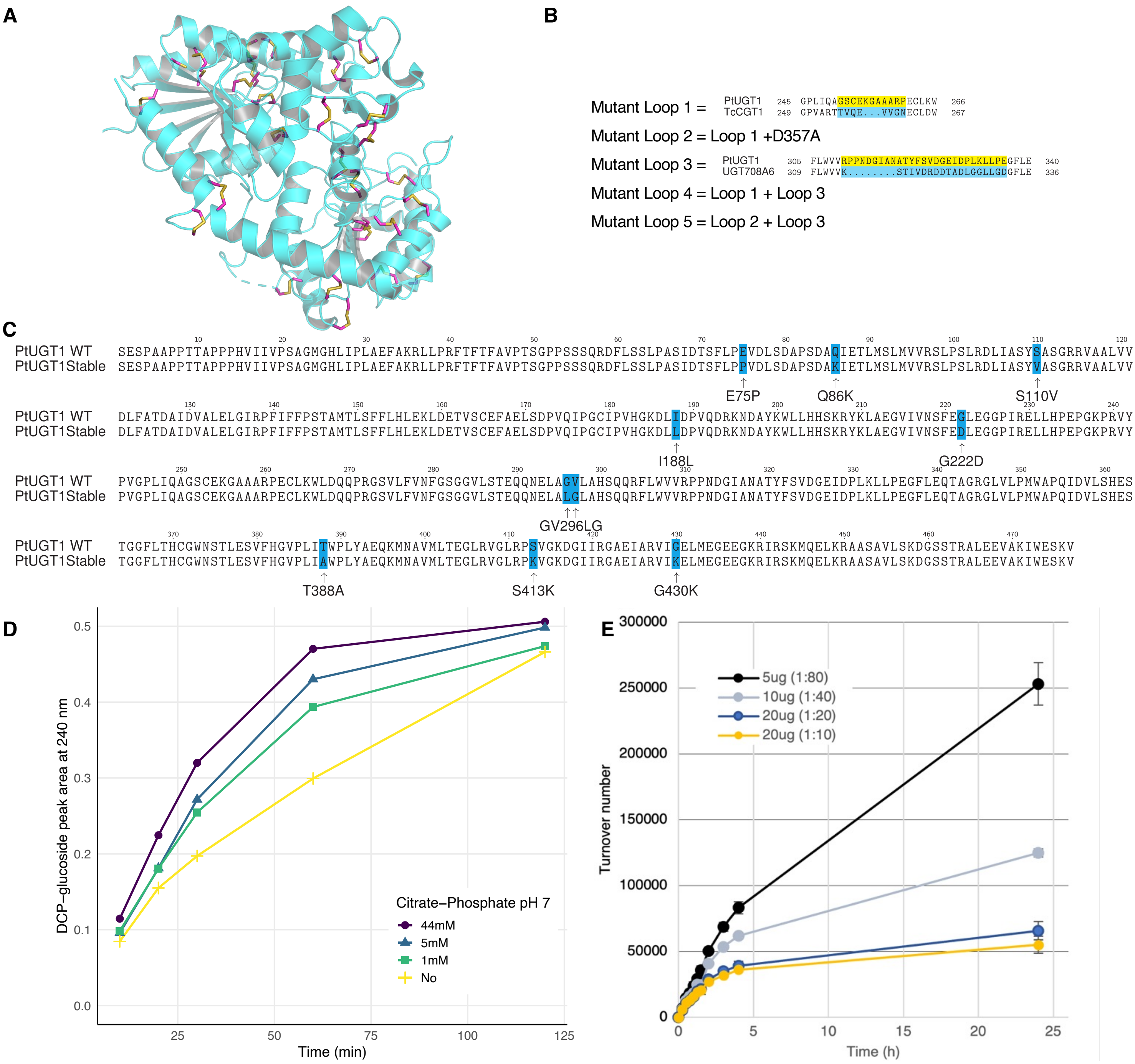

Supplementary Figure 1. Rational stability engineering of PtUGT1 and process characterization. (**A**) Cartoon representation of *Pt*UGT1 (PDB ID 5nlm), highlighted with residue pairs that hypothetically could form intramolecular disulfide bridges if mutated to cysteines (yellow and magenta sticks). (**B**) Loop sequences grafted (blue) to replace *Pt*UGT1 loops (yellow). Mutations shown in panels A and B are not present in *Pt*UGT1^{stable} (Mut 87, Supplementary Table 1). (**C**) Sequence alignment of *Pt*UGT1 and *Pt*UGT1^{stable}, annotated with mutations from consensus mutagenesis (blue). (**D**) Effect of buffer minimization on product formation by *Pt*UGT1^{stable}.Curves correspond to reactions performed in 44mM (purple), 5mM (blue), 1mM (green) citrate-phosphate pH 7, or without buffer (yellow). The reaction is slower with reduced buffer but reaches similar levels of completion. (**E**) Turnover numbers of *Pt*UGT1^{stable} on indoxyl. The total TON cannot be absolutely determined, since the reactions reached completion, but it is at least 250,000. The curves represent varying enzyme amounts, either 5μg (black), 10μg (grey), or 20μg (blue and yellow) of PtUGT1^{stable}, and the values in parentheses denote *Pt*UGT1^{stable}:SuSy ratio. The blue and the yellow curves demonstrate that the reaction is not limited by SuSy. Data presented are mean ± SD n=2 . Source data are provided as a Source Data file.

A

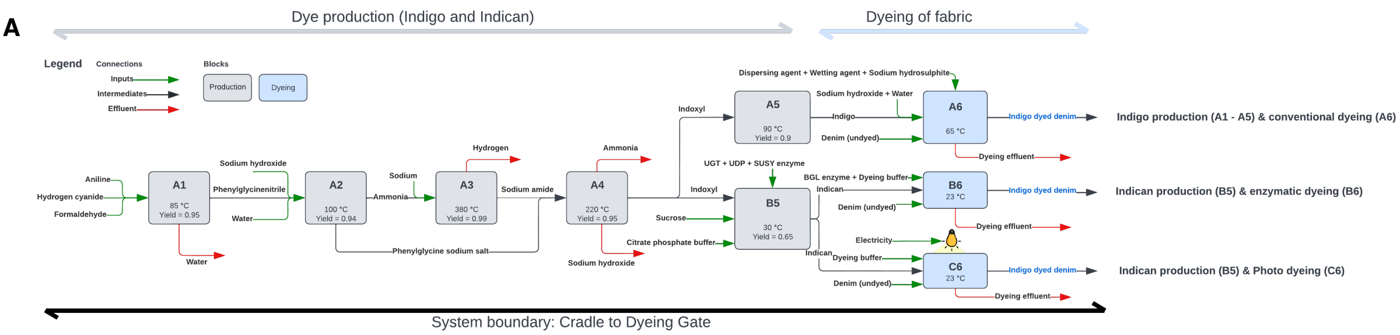

B

Amounts to produce 1 kg of indican				
Component	Preliminary experiment	Theoretically optimized	Validation experiment	Theoretical industrial process
Indoxyl (mM)	1.75	100	100	100
UGT (g/L)	1.57	1.57	0.250	0.250
UDP-glc (mM)	5.00	0	0	0
Water (L)	1935	52		52.0
Citrate phosphate 90 mM (L)	0	0	52.0	0
Hepes (mM)	44.0	0	0	0
SuSy (g/L)	0	0.00850	2.16	2.16
Sucrose (mM)	0	100	200	200
UDP (mM)	0	0.500	1.00	1.00
Conversion (%)	100	60.0	65.0	65.0

Costs to produce 1 kg of indican (USD)				
Component	Preliminary experiment	Theoretically optimized	Validation experiment	Theoretical industrial process
Indoxyl	2.25	3.76	3.47	3.47
UGT	2.22	2.22	0.326	0.33
UDP-glc	465199	0	0.00	0.00
Water	14.9	0.4	0.00	0.40
Citrate phosphate 90 mM	0	0	5.38	0.00
Hepes	5001	0	0.00	0.00
SuSy	0	0.0120	2.81	2.81
Sucrose	0	0.966	1.78	1.78
UDP	0	0.570	1.05	1.06
Total	470220	7.92	14.8	9.85

Amounts to dye one pair of jeans (excl. dyestuff)				
Component	Conventional dyeing	Enzymatic dyeing	Bulb photolytic dyeing	LED photolytic dyeing
Dispersing agent (Setamol) (kg)	0.000387	0	0	0
Wetting agent (kg)	0.000100	0	0	0
Caustic soda (27 weight%)	0.0231	0	0	0
Sodium hydrosulphite (kg)	0.00808	0	0	0
Water (L)	1.63	0	11.23	11.23
Phosphate buffer 20 mM (L)	0	3.18	0	0
β-glucosidase (g)	0	0.500	0	0
30% HCl (aq) (kg)	0	0.0	0.0250	0.0250
Electricity (kWh)	n/a	n/a	2250	0.400

Costs to dye one pair of jeans (USD) (excl. dyestuff)				
Component	Conventional dyeing	Enzymatic dyeing	Bulb photolytic dyeing	LED photolytic dyeing
Dispersing agent (Setamol)	0.0000154	0	0	0
Wetting agent	0.0000090	0	0	0
Caustic soda (27 weight%)	0.104	0	0	0
Sodium hydrosulphite	0.00929	0	0	0
Water	0.0126	0	0.0865	0.0865
Phosphate buffer 20 mM	0	0.188	0	0
β-glucosidase	0	0.0125	0	0
30% HCl (aq)			0.00150	0.00150
Electricity			239	0.0424
Total	0.126	0.200	239	0.130

Supplementary Figure 2. Techno-economic assessment.

(**A**) Process flow diagram for the conventional, enzymatic, and photolytic processes. The arrows represent flow of input material (green), intermediates (black), or effluents (red). (**B**) Raw material cost tables. Raw material costs associated with indigo/indican production were calculated for four scenarios: the preliminary laboratory experiment; a theoretical optimization; the laboratory experiment to validate the theoretically optimized scenario; and a theoretical industrial process with pH control. Raw material costs relating to dyeing are for one dyeing cycle. All assumptions and data sources for TEA are summarized in Supplementary Table 3.

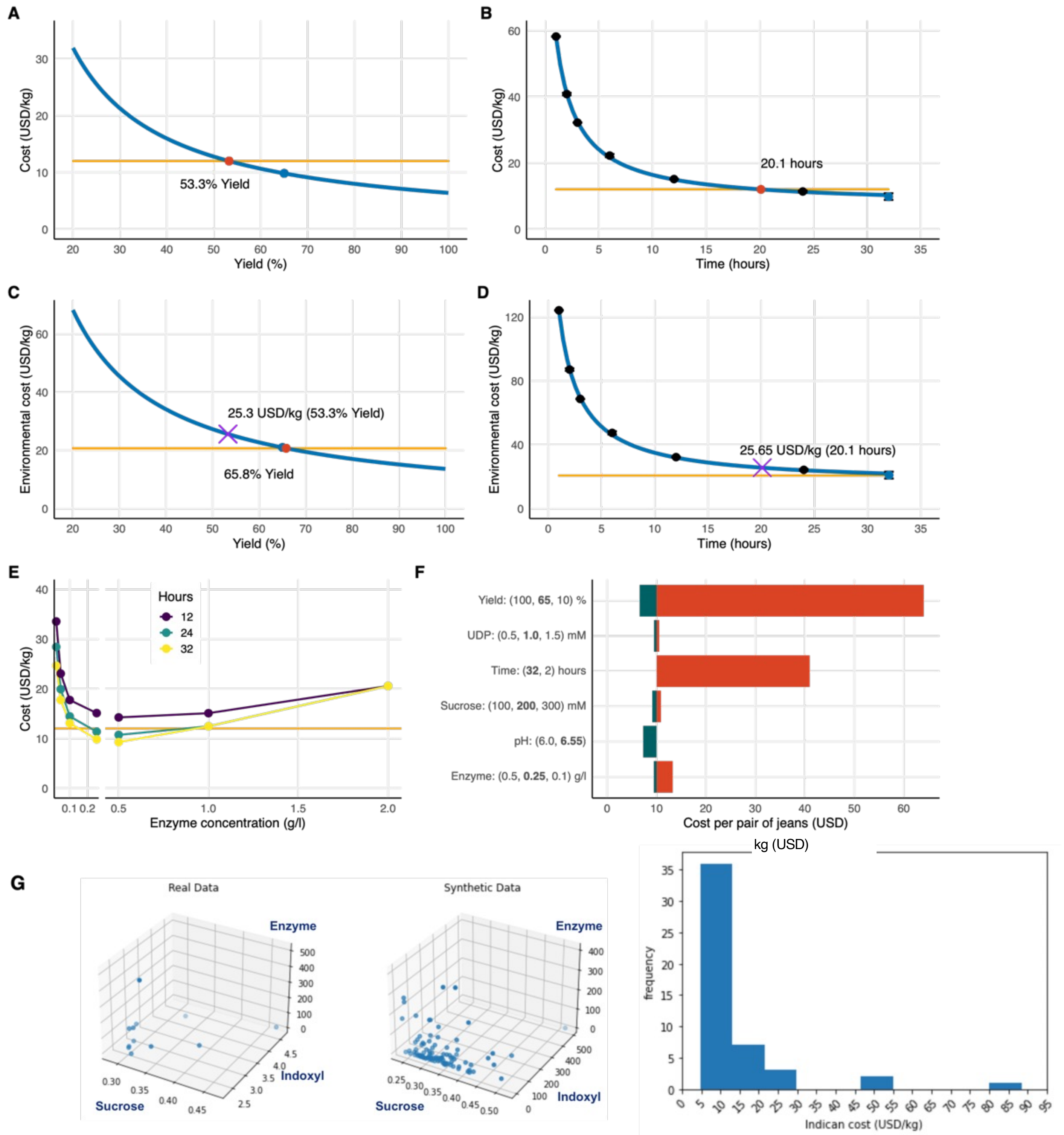

Supplementary Figure 3. Sensitivity and uncertainty analyses for production of indican.

(**A-D**) Graphs are based on experimental data presented in Fig. 2B. The orange line represents economic feasibility (12 USD/kg) (A-B) or environmental cost competitiveness (20 USD/kg) (C-D). Experimental data points are depicted as black dots, the baseline condition is shown as a blue dot, and the intersection with the target value as a red dot. Purple crosses indicate the cut-off for economic feasibility . (**E**) A projection of cost resulting from increasing enzyme amounts is shown, extrapolated from data in Fig. 2B (data from 0.5 g/l are projected). The curves correspond to different reaction lengths, either 12 (purple), 24 (green), or 32 (yellow) hours. (**F**) The variation in cost resulting from varying process parameters and cost-driving reactants (+/- 50%) is summarized using green bars (reductions) or red bars (increases). The actual parameter values is shown in bold. 0.5 mM UDP and 100 mM sucrose will likely be functional[1]. Assuming that SuSy is still limiting, a slight decrease in cost could potentially be achieved by tuning pH to 6.0, where SuSy is more active[1]. (**G**) Uncertainty analysis on the selling prices of the three cost-driving input materials. Source data are provided as a Source Data file.

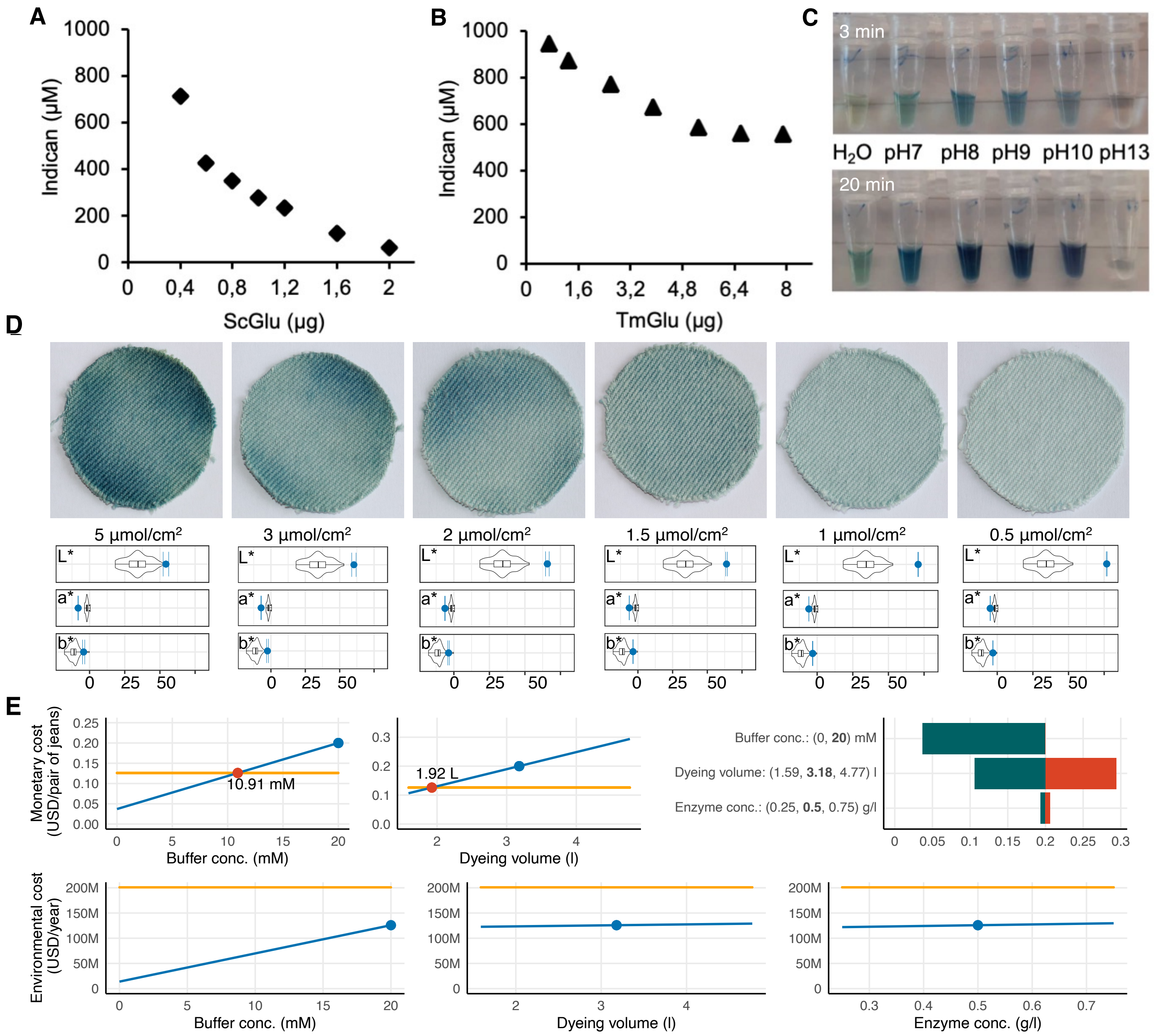

Supplementary Figure 4. Enzymatic indican dyeing

(**A-B**) Scatter graph showing hydrolysis of indican by beta-glucosidases (**C**) Indigo formation from indican by *Sc*Glu is optimal at pH 9. (**D**) Denim swatches dyed with *Sc*Glu and varying amounts of indican. CIELAB color space values (L* = dark-light, a* = green-red, b* = blue-yellow) shown below each swatch are presented as mean ± SD n=5 (5 positions within each swatch), confirming that indican amount can control the darkness of the color (L*), while the green-red and blue-yellow balances remain unchanged. Values found in a selection of 60 samples of commercial blue denim (Supplementary Fig. 7) are shown as violin and box plots for comparison. Center lines show the medians; box limits indicate the 25th and 75th percentiles as determined by R software; whiskers extend 1.5 times the interquartile range from the 25th and 75th percentiles, outliers are represented by dots. Source data are provided as a Source Data file. (**E**) Sensitivity analyses. The orange line represents economic feasibility (0.14 USD/pair of jeans) (upper two plots) or environmental cost competitiveness with the other presented technology, photolytic dyeing (201 M USD/year) (lower three plots). The baseline condition is shown as a blue dot, and the intersection with the target value as a red dot. In the upper right is an overview of varying the three components. Like dyeing volume, beta-glucosidase is varied +/- 50%. Buffer and dyeing volume are cost drivers, with a potential to significantly reduce the cost. The actual parameter values is shown in bold. The analysis assumes that dyeing efficiency is not compromised by varying these three process parameters.

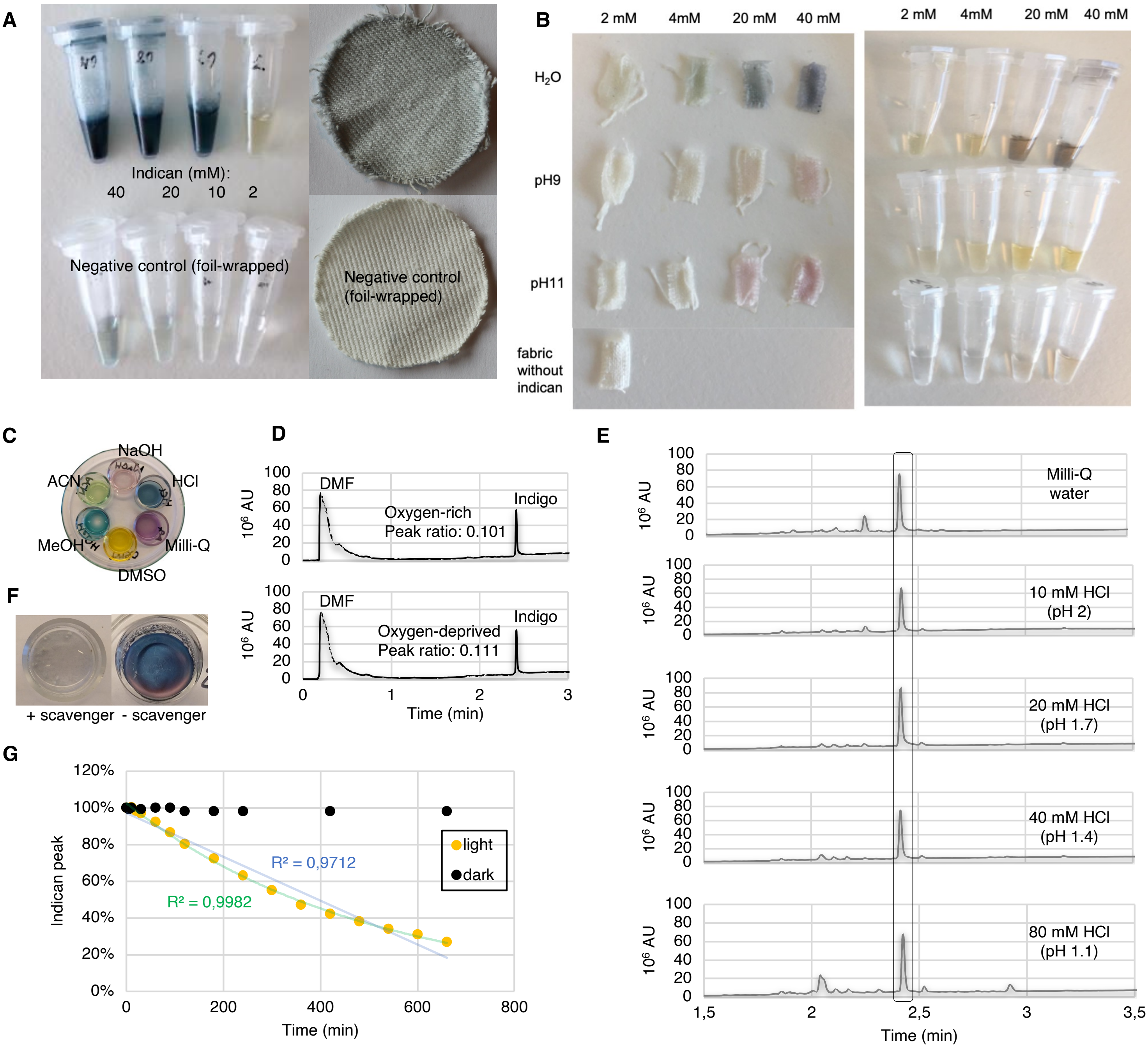

Supplementary Figure 5. Discovery and characterization of a photolytic indigo-forming reaction. (**A**) Natural sunlight promotes indigo formation from indican in solution and on textile. (**B**) A household light bulb promotes indigo formation from indican, in solution and on textile. (**C**) photolytic cleavage of indican in different solvents gives rise to a variety of colors, with HCl promoting the expected indigo-blue color. (**D**) Photolytic indigo formation from indican seems to be independent of oxygen. (**E**) Varying pH with HCl between 1 and 2 identified pH 1.7 (20 mM HCl (aq)) as optimal. The annotated peak was confirmed to be indigo by NMR (Supplementary Fig 8A), and color extracted from commercial denim gave rise to a peak with identical retention time (Supplementary Fig 8B). (**F**) Gallic acid – a radical scavenger - quenches the reaction. Excess gallic acid solids can be seen in the sample. (**G**) Photolytic indican cleavage under aqueous HCl conditions (pH 1.7) displays first order reaction kinetics. No indican degradation is detected in the dark under the same aqueous 20 mM HCl conditions (pH 1.7), demonstrating that the reaction is catalyzed by the light.

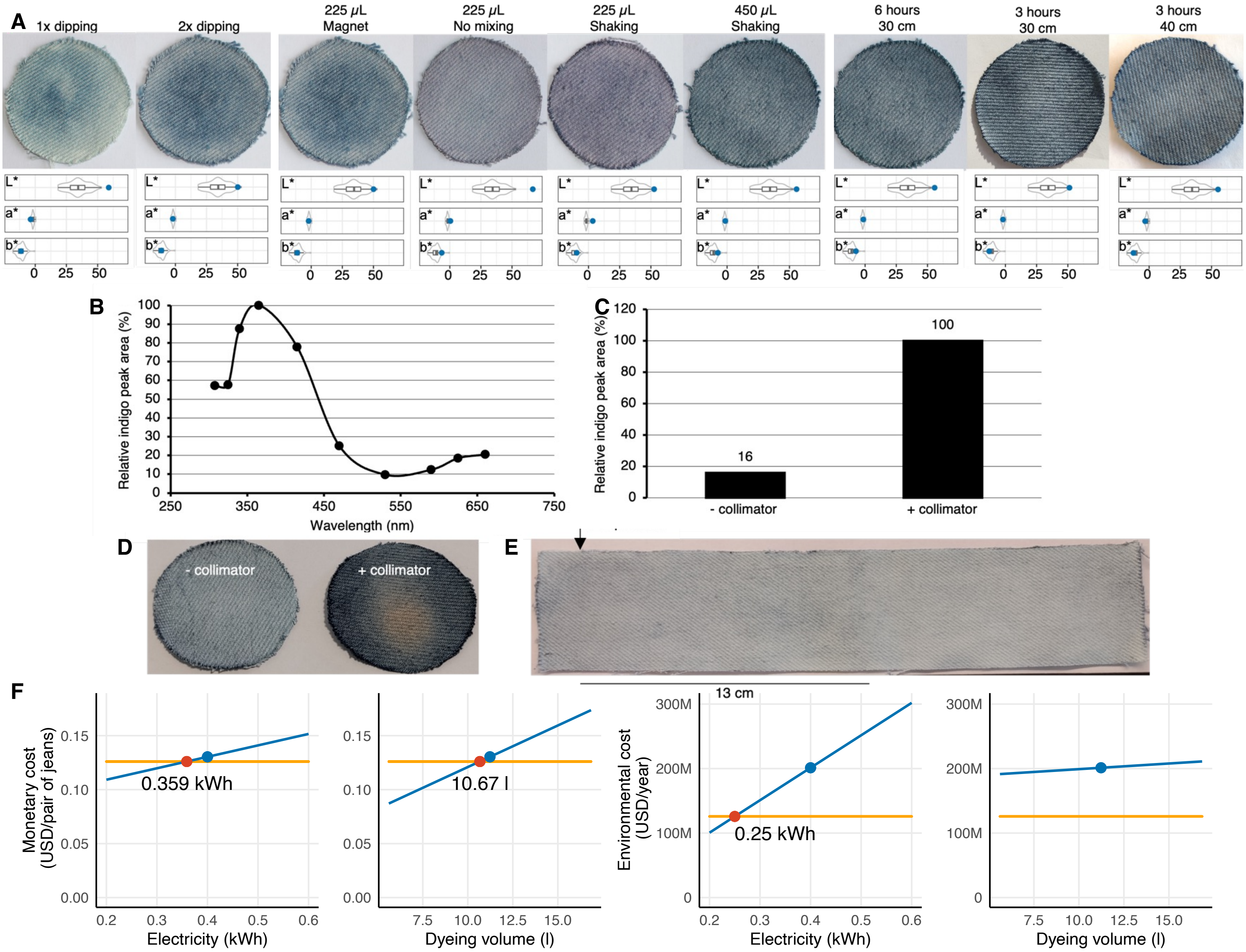

Supplementary Figure 6. Photolytic indican dyeing

(**A**) Dyeing with an artificial sunlight bulb. CIELAB color space values (L* = dark-light, a* = green-red, b* = blue-yellow) shown below each swatch are presented as mean ± SD n=5 (5 positions within each swatch). Values found in a selection of 60 samples of commercial blue denim (Supplementary Fig. 7) shown as violin and box plots for comparison. Center lines show the medians; box limits indicate the 25th and 75th percentiles as determined by R software; whiskers extend 1.5 times the interquartile range from the 25th and 75th percentiles, outliers are represented by dots. Shaking gives rise to even and efficient color development but promotes byproduct formation (red, see a* value, likely due to excess aeration), which can be alleviated by increasing the liquid volume, which is given here per square centimeter of fabric. Decreasing the exposure time to half and increasing lamp distance to allow a larger area to be illuminated did not compromise color quality. (**B-E**) Dyeing with LED. (**B**) Wavelength dependency of indigo formation. (**C**) Indigo formation from indican by a LED at 365 nm with and without a collimator for adjusting the light into a parallel beam that fits the sample. (**D**) Dyeing with a LED at 365 nm with and without a collimator. With the collimator, the lamp is too close (14 cm), causing miscoloring at the center. (**E**) Establishment of effective dyeing radius. The lamp distance is 50 cm. There is even coloring up to 13 cm from the LED, after which there is a visual decrease in color intensity. (**F**) Sensitivity analyses. Monetary cost (blue lines) depending on dyeing volume or electricity usage, varied +/- 50% from the lab-scale parameters. The orange lines represent economic feasibility (0.14 USD/pair of jeans) (first two plots) or environmental cost competitiveness with the other presented technology, enzymatic dyeing (126 M USD/year) (last two plots). The baseline condition is shown as a blue dot, and the intersection with the target value as a red dot.

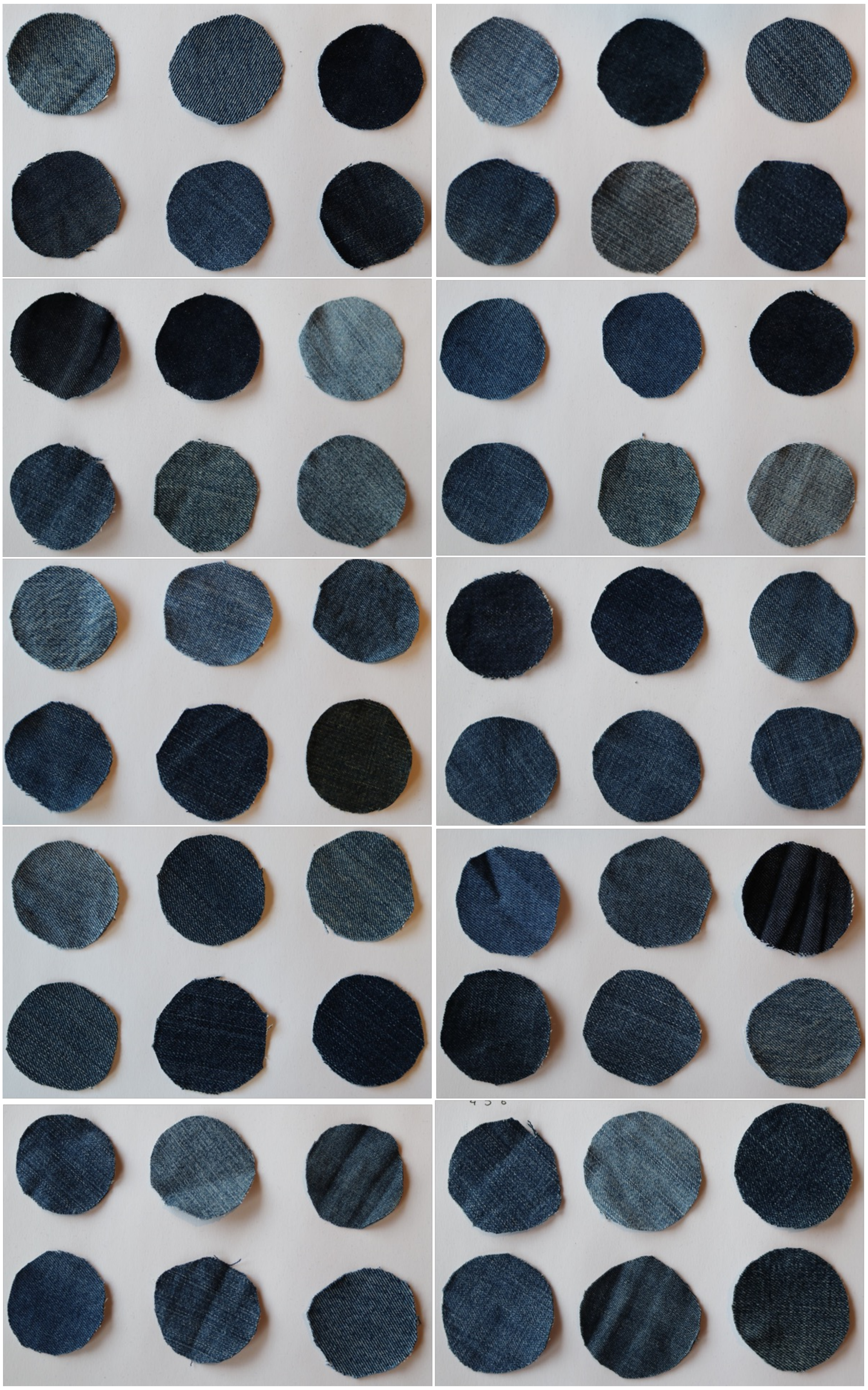

Supplementary Figure 7. 60 samples of commercial dark blue denim. These were used as a reference when evaluating laboratory-dyed swatches, since we did not have access to dyed denim before industrial washing.

A

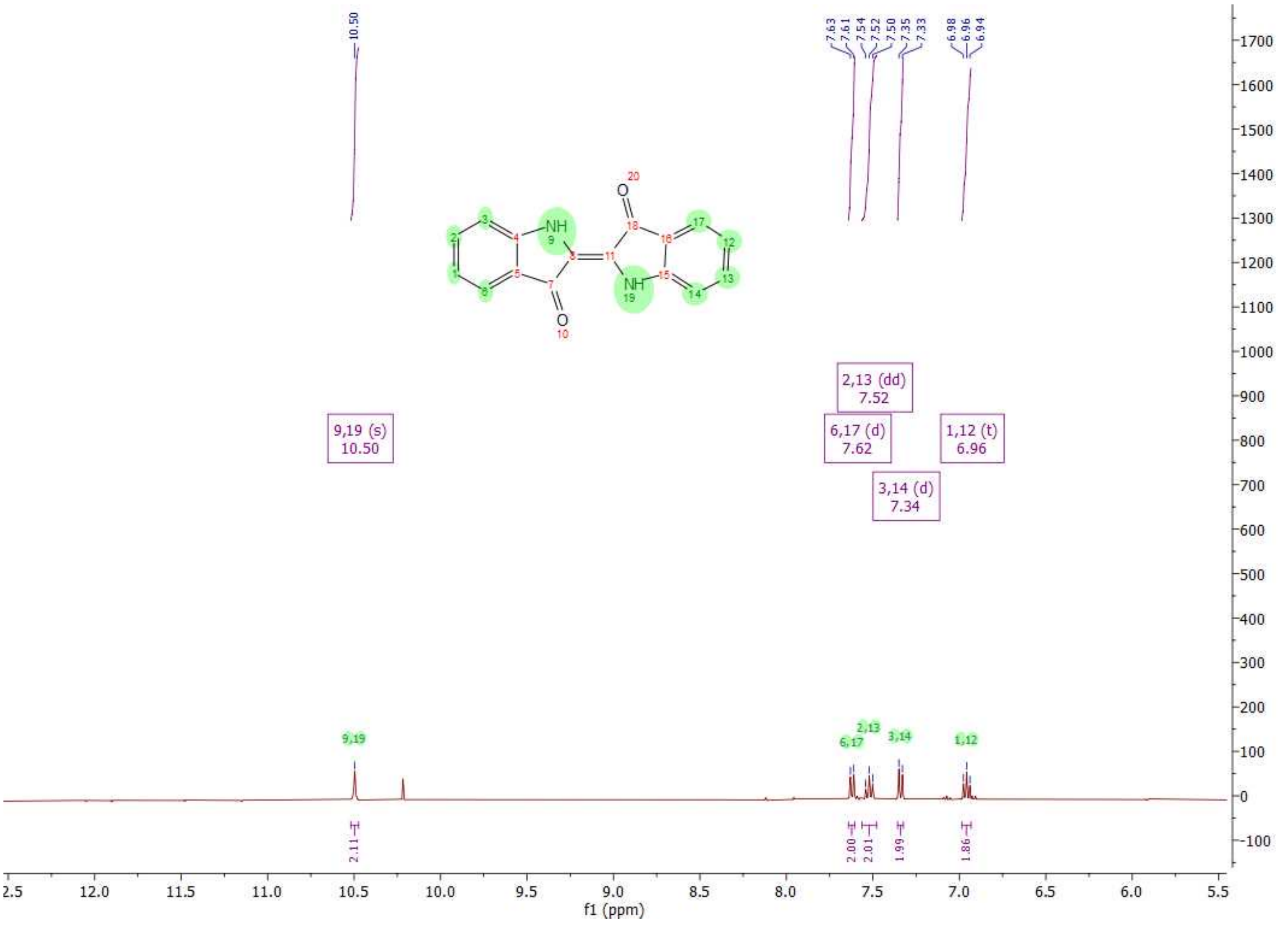

B

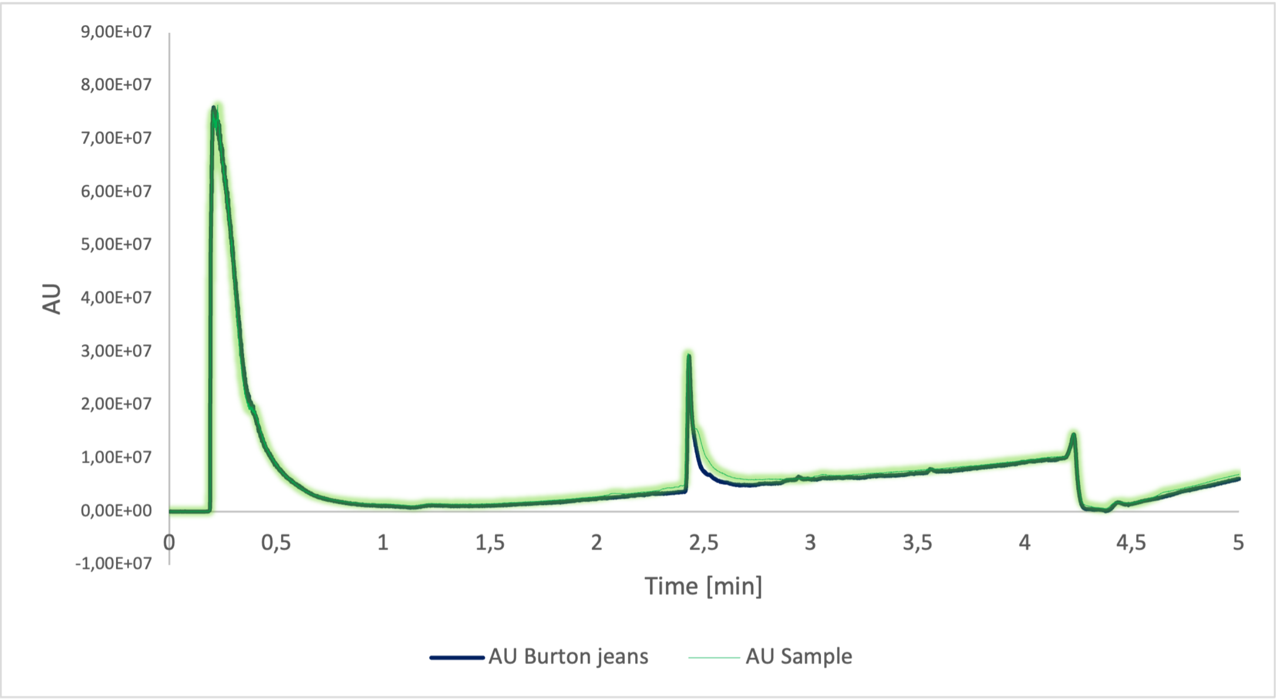

Supplementary Figure 8. Characterization of photolytically formed indigo.

(**A**) Zoomed-in area of indigo peaks within the proton NMR. ^{1}H NMR (400 MHz, DMSO) δ 10.50 (s, 2H), 7.62 (d, J = 7.5 Hz, 2H), 7.52 (dd, J = 7.9, 7.5 Hz, 2H), 7.34 (d, J = 7.9 Hz, 2H), 6.96 (t, J = 7.5 Hz, 2H). (**B**) LC-MS of indigo extracted from commercial denim (blue) compared with photolytically formed indigo (green). Source data are provided as a Source Data file-

Supplementary Table 1: List of *Pt*UGT1 variants and their relative T_m and activity.

Mutant ID*	mutations	ΔTmB (°C)§	Rel. activity (%)†
1	P14C, A117C	-1.42 ± 0.02	
2	S21C, D122C	-1.1 ± 0.04	
4	L119C, A132C	-1.13 ± 0.04	
5	V121C, A125C	-0.75 ± 0.08	
6	T126C, R208C	-2.59 ± 0.15	
7	A132C, I137C	-1.65 ± 0.06	
8	A132C, P139C	-2.97 ± 0.02	
9	A147C, P227C	0.09 ± 0.04	
10	P173C, P181C	-3.21 ± 0.58	
11	I176C	-1.67 ± 0.02	
12	D186C, K396C	-0.31 ± 0.02	
13	P190C, A198C	1.96 ± 0.07	32.27 ± 3.61
14	D193C, N196C	0.53 ± 0.04	
15	A259C, L264C	-1.75 ± 0.02	
16	P271C, S274C	-2.33 ± 0.03	
17	G273C, G365C	-0.66 ± 0.05	
18	V278C, W307C	-4.51 ± 0.54	
19	L286C, Q290C	-4.91 ± 0.05	
20	G337C, E340C	-1.55 ± 0.02	
21	T342C, G346C	-4.75 ± 0.06	
22	L368C, I387C	-6.79 ± 0.08	
23	T388C, A399C	2.54 ± 0.02	64.52 ± 2.52
24	P412C, E424C	-5.71 ± 0.06	
25	V455C, S462C	-5.93 ± 0.02	
26	S457C, G460C	-5.13 ± 0.02	
27	L64C, I68C	-4.78 ± 0.05	
28	T146C, M148C	4.17 ± 0.04	76.97 ± 0.63
29	A147C, E224C	-6.59 ± 0.52	
30	E235C, K238C	-2.05 ± 0.02	
31	L267C, S363C	-2.5 ± 0.03	
32	N279C, V308C	-1.28 ± 0.01	
33	P390C, Q395C	-1.96 ± 0.09	
34	G460C, T463C	-8.93 ± 0.09	
35	P48C, S98C	-2.22 ± 0.03	
36	A66C, P13C	-0.65 ± 0.05	
37	S112C, T10C	-0.93 ± 0.08	
13+23	P190C, A198C, T388C, A399C	8.36 ± 0.12	43.27 ± 1.64
13+28	P190C, A198C, T146C, M148C	8.24 ± 0.18	29.23 ± 3.17
23+28	T388C, A399C, T146C, M148C	12.96 ± 0.18	18.22 ± 3.29
13+23+28	P190C, A198C, T388C, A399C, T146C, M148C	16.49 ± 0.21	0
38	A22P, M91I	0.22 ± 0.28	
39	E75P	1.42 ± 0.17	105.02 ± 1.72
40	E157P	0.26 ± 0.22	
41	G222D	2.35 ± 0.18	72.38 ± 3.56
42	G405D	0.42 ± 0.1	
43	G409A	0.39 ± 0.15	
44	G430K	3.5 ± 0.1	104.87 ± 2.90
45	G222D, G405D, G409A, G430K	3.45 ± 0.18	
46	E75P, E157P, G222D, G405D, G409A, G430K	5.07 ± 0.08	
47	S50D, M94T	-2.03 ± 0.11	
48	Q86K	2.36 ± 0.1	99.11 ± 4.54
48B	Q86R	2.31 ± 0.11	54.16 ± 4.22
49	A107K	-0.37 ± 0.06	
50	K210R	-0.56 ± 0.13	
51	Q269E	-0.75 ± 0.19	
52	V285T	0.11 ± 0.2	
53	A299E, Q341R	-0.55 ± 0.65	
54	K332D, E336K, Q341R, A343K	-0.32 ± 0.05	
55	S413K	0.75 ± 0.08	100.83 ± 1.72
56	I472K	-2.16 ± 0.22	
57	A81L	0.41 ± 0.12	
58	S110V	1.17 ± 0.13	104.22 ± 1.76
59	I129F	-1.79 ± 0.36	
60	I188L	1.09 ± 0.35	132.60 ± 0.88
61	L333F	0.48 ± 0.37	
62	M351S	-0.81 ± 0.57	
63	F381V	2.89 ± 0.07	74.12 ± 3.39
64	T388A	0.86 ± 0.17	99.35 ± 2.64
65	S55A	-0.16 ± 0.13	
66	A125G	0.44 ± 0.09	
67	F140Y, I472K	-1.49 ± 0.26	
68	GV296/297LG	6.02 ± 0.27	59.36 ± 1.23
69	H300M	-0.83 ± 0.3	
70	E340G	-0.4 ± 0.29	
Loop 1	GSCEKGAAARP 251-261 to VQEVVGN	-7.43 ± 0.01	
Loop 2	GSCEKGAAARP 251-261 to VQEVVGN + D357A	-3.63 ± 0.01	
Loop 3	RPPNDGIANATYFSVDGEIDPLKLL PE 332-358 to KSTIVDRDDTADLGGLLGD	-5.16 ± 0.06	
Loop 4	Loop 1 + Loop 3	-2.23 ± 0.1	
Loop 5	Loop 2 + Loop3	-2.6 ± 0.55	
	Combination of mutants		
71	Q86K, S110V	2.82 ± 0.07	101.98 ± 3.30
72	S413K, T388A	1.8 ± 0.13	127.04 ± 6.59
73	F381V, GV296/297LG	6.99 ± 0.05	67.82 ± 2.87
74	Q86K, S110V, I188L, T388A	4.61 ± 0.1	158.97 ± 1.29
75	S413K, T388A, I188L	3.49 ± 0.12	162.61 ± 3.46
76	F381V, GV296/297LG, I188L	7.4 ± 0.25	132.38 ± 2.05
77	Q86K, S110V, I188L, S413K	5.88 ± 0.21	167.56 ± 4.00
78	Q86K, S110V, I188L	4.88 ± 0.07	190.24 ± 2.40
79	F381V, GV296/297LG, I188L, G430K	7.82 ± 0.1	118.50 ± 2.90
80	Q86K, S110V, I188L, S413K, T388A	6.03 ± 0.06	179.93 ± 4.67
81	Q86K, S110V, I188L, S413K, T388A, G430K	6.76 ± 0.08	184.74 ± 2.96
82	E75P, G430K	2.07 ± 0.17	115.71 ± 4.89
83	E75P, G222D	0.33 ± 0.17	53.77 ± 2.65
84	E75P, G222D, G430K	2.68 ± 0.18	74.25 ± 2.28
85	Q86K, S110V, I188L, S413K, T388A, G430K, E75P	7.83 ± 0.11	217.60 ± 2.70
86	Q86K, S110V, I188L, S413K, T388A, G430K, E75P, G222D	8.92 ± 0.08	180.53 ± 4.77
87 (PtUGT1^{stable})	Q86K, S110V, I188L, S413K, T388A, G430K, E75P, G222D, GV296/297LG	13.14 ± 0.07	105.46 ± 2.85
88	Q86K, S110V, I188L, S413K, T388A, G430K, E75P, G222D, GV296/297LG, F381V	13.34 ± 0.06	100.00 ± 2.89
89	Q86K, S110V, I188L, S413K, T388A, G430K, E75P, G222D, GV296/297LG, F381V, T146C, M148C	14.14 ± 0.22	86.00 ± 3.06
90	Q86K, S110V, I188L, S413K, G430K, E75P, G222D, GV296/297LG, F381V, T388C, A399C	15.64 ± 0.08	89.86 ± 1.60
91	E75P, G430K, Q86K	4.73 ± 0.2	126.00 ± 2.01
92	E75P, G430K, Q86K, GV296/297LG	8.48 ± 0.22	62.52 ± 1.01
93	E75P, G430K, Q86K, T388C, A399C	7.43 ± 0.15	129.11 ± 4.38
94	E75P, G430K, Q86K, T146C, M148C	6.42 ± 0.13	66.67 ± 6.16
96	Q86K, S110V, I188L, S413K, G430K, E75P, G222D, T388C, A399C	10.38 ± 0.13	170.70 ± 16.72
97	Q86K, S110V, I188L, S413K, T388A, G430K, E75P, G222D, T146C, M148C	9.62 ± 0.15	66.88 ± 16.53
98	Q86K, S110V, I188L, S413K, G430K, E75P, G222D, GV296/297LG, T388C, A399C	14.32 ± 0.07	103.05 ± 3.21
99	Q86K, S110V, I188L, S413K, T388A, G430K, E75P, G222D, GV296/297LG, T146C, M148C	12.45 ± 0.12	83.65 ± 1.07

* Beneficial mutations combined in further rounds of optimization according to figure 2 in the main text are highlighted in green.

Data are mean values n=3.

§ΔTmB (°C) ± Standard error of mean (SEM) is given using as reference TmB of PtUGT1 WT.

† Activity is measured relative to wildtype activity, at 40 °C ± Standard deviation.

Supplementary Table 2: LCA.

**Midpoints**	Production (1 kg)						Dyeing (1 pair of jeans)‡			
	Indigo and indican shared process steps†				Indigo	Indican	Indigo	*Sc*Glu	sunlight	LED
Impact category (equivalents)*	A1	A2	A3	A4	A5	B5	A6	B6	C6	C6
Global warming (kg CO_2)	6.72E+00	4.28E+01	1.50E+00	1.02E-05	4.95E-06	6.45E-01	9.86E-01	4.39E-02	3.83E+00	1.66E-01
Stratospheric ozone depletion (kg CFC-11)	6.30E-05	9.23E-06	5.18E-07	2.62E-12	1.27E-12	3.08E-06	2.92E-07	9.10E-08	2.17E-06	9.60E-08
Ionizing radiation (kBq ^{60}Co)	1.08E-01	4.37E-01	5.97E-02	2.93E-07	1.42E-07	0.09E-01	2.88E-02	1.37E-03	1.93E+00	8.11E-02
Ozone formation, human health (kg NOx)	1.54E-02	5.74E-02	2.82E-03	2.88E-08	1.39E-08	2.10E-03	1.28E-03	1.08E-04	6.97E-03	3.06E-04
Fine particulate matter formation (kg PM2.5)	1.10E-02	4.28E-02	2.41E-03	1.81E-08	8.80E-09	1.43E-03	8.68E-04	1.08E-04	5.78E-03	2.56E-04
Ozone formation, terrestrial ecosystems (kg NOx)	1.64E-02	5.86E-02	2.88E-03	2.97E-08	1.44E-08	2.14E-03	1.32E-03	1.09E-04	7.03E-03	3.09E-04
Terrestrial acidification (kg SO_2)	2.93E-02	1.13E-01	4.94E-03	2.87E-08	1.39E-08	5.64E-03	2.42E-03	3.27E-04	1.46E-02	6.45E-04
Freshwater eutrophication (kg P)	1.59E-03	5.33E-03	3.95E-04	2.65E-09	1.28E-09	4.09E-04	1.22E-04	1.70E-05	3.74E-03	1.59E-04
Marine eutrophication (kg N)	9.42E-04	3.74E-04	5.28E-05	3.77E-10	1.83E-10	8.94E-04	9.43E-06	2.15E-05	2.65E-04	1.13E-05
Terrestrial ecotoxicity (kg 1.4-DCB)	1.95E+01	1.49E+02	7.99E+00	7.36E-05	3.57E-05	3.98E+00	1.66E+00	1.68E-01	4.29E+00	2.09E-01
Freshwater ecotoxicity (kg 1.4-DCB)	3.34E-01	3.41E-01	8.06E-02	2.11E-05	1.02E-05	5.83E-02	9.65E-03	3.13E-03	1.67E-01	7.52E-03
Marine ecotoxicity (kg 1.4-DCB)	4.27E-01	5.26E-01	1.06E-01	2.64E-05	1.28E-05	6.07E-02	1.43E-02	3.86E-03	2.22E-01	9.98E-03
Human carcinogenic toxicity (kg 1.4-DCB)	2.06E-01	4.80E-01	4.34E-02	3.52E-05	1.70E-05	3.23E-02	1.20E-02	2.07E-03	2.23E-01	9.74E-03
Human non-carcinogenic toxicity (kg 1.4-DCB)	6.83E+00	1.42E+01	1.42E+00	3.75E-04	1.82E-04	1.42E+00	2.58E-01	5.44E-02	4.95E+00	2.17E-01
Land use (m^2 crop)	7.11E-02	1.81E-01	2.10E-02	2.86E-07	1.39E-07	4.22E-01	1.06E-02	1.08E-02	1.39E-01	5.95E-03
Mineral resource scarcity (kg Cu)	5.26E-02	1.39E-02	5.33E-03	2.70E-07	1.31E-07	5.08E-03	5.88E-04	4.11E-04	4.98E-03	3.18E-04
Fossil resource scarcity (kg oil)	2.67E+00	1.21E+01	4.73E-01	2.64E-06	1.28E-06	1.55E-01	3.28E-01	9.40E-03	1.01E+00	4.38E-02
Water consumption (m^3)	7.54E-02	6.73E-02	2.66E-02	1.28E-07	6.20E-08	1.32E-01	4.83E-03	5.69E-03	7.69E-02	1.42E-02

**Endpoints, production (1 kg)**							**Endpoints, dyeing (1 pair of jeans)** ‡		
		Indigo			Indican			Indigo	
Impact category	Total	Sum	Monetary (USD)	Total	Sum	Monetary (USD)[2]	Total	Sum	Monetary (USD)[2]
Global warming. Human health (DALY)	4.73E-05	1.57E-04	1.57E+01	4.79E-05	1.59E-04	1.59E+01	4,88E-07	1,62E-06	1,62E-01
Stratospheric ozone depletion (DALY)	3.86E-08			4.02E-08			3,98E-10		
Ionizing radiation (DALY)	5.14E-09			5.92E-09			5,30E-11		
Ozone formation, Human health (DALY)	6.88E-08			7.07E-08			7,09E-10		
Fine particulate matter formation (DALY)	1.01E-04			1.02E-04			1,05E-06		
Human carcinogenic toxicity (DALY)	2.42E-06			2.53E-06			2,49E-08		
Human non-carcinogenic toxicity (DALY)	5.11E-06			5.44E-06			5,27E-08		
Water consumption, Human health (DALY)	3.16E-07			4.71E-07			3,88E-09		
Global warming. Terr ecosyst (species.yr)	1.43E-07	3.78E-07	2.46E-02	1.45E-07	3.87E-07	2.51E-02	1,47E-09	3,90E-09	2,54E-04
Global warming, Freshwat ecosyst (species.yr)	3.90E-12			3.95E-12			4,02E-14		
Ozone formation, Terr ecosyst (species.yr)	1.00E-08			1.03E-08			1,04E-10		
Terrestrial acidification (species.yr)	2.13E-07			2.15E-07			2,20E-09		
Freshwater eutrophication (species.yr)	4.90E-09			5.17E-09			5,05E-11		
Marine eutrophication (species.yr)	2.33E-12			3.85E-12			2,40E-14		
Terrestrial ecotoxicity (species.yr)	2.01E-09			2.05E-09			2,07E-11		
Freshwater ecotoxicity (species.yr)	5.23E-10			5.63E-10			5,41E-12		
Marine ecotoxicity (species.yr)	1.11E-10			1.18E-10			1,15E-12		
Land use (species.yr)	2.42E-09			6.17E-09			2,50E-11		
Water consumption Terr ecosyst (species.yr)	2.08E-09			3.19E-09			2,36E-11		
Water consumption, Aqua ecosyst (species.yr)	2.08E-13			3.62E-13			1,05E-15		
Mineral resource scarcity (USD2013)	1.66E-02	5.05E+00	5.05E+00	1.78E-02	5.09E+00	5.09E+00	1,70E-04	5,20E-02	5,20E-02
Fossil resource scarcity (USD2013)	5.03E+00			5.08E+00			5,18E-02		
Total monetary (USD)			20.7E+00			21.0E+00			2,14E-01

**Endpoints for dyeing (1 pair of jeans) (cont.)**		*Sc*Glu			Sunlight bulb			LED	
Impact category	Total	Sum	Monetary (USD)[2]	Total	Sum	Monetary (USD)[2]	Total	Sum	Monetary (USD)[2]
Global warming, Human health (DALY)	4,08E-08	1,40E-07	1,40E-02	3.55E-06	9.25E-06	9.25E-01	1.54E-07	4.29E-07	4.29E-02
Stratospheric ozone depletion (DALY)	4,83E-11			1.15E-09			5.10E-11		
Ionizing radiation (DALY)	1,17E-11			1.64E-08			6.88E-10		
Ozone formation, Human health (DALY)	9,82E-11			6.34E-09			2.78E-10		
Fine particulate matter formation (DALY)	6,76E-08			3.63E-06			1.61E-07		
Human carcinogenic toxicity (DALY)	6,89E-09			7.41E-07			3.23E-08		
Human non-carcinogenic toxicity (DALY)	1,24E-08			1.13E-06			4.94E-08		
Water consumption, Human health (DALY)	1,26E-08			1,71E-07			3,15E-08		
Global warming, Terrestrial ecosyst (species.yr)	1,23E-10	3,95E-10	2,57E-05	1.07E-08	1.97E-08	1.28E-03	4.64E-10	1,00E-09	6,50E-05
Global warming, Freshwater ecosyst (species.yr)	3,36E-15			2.93E-13			1.27E-14		
Ozone formation, Terrestrial ecosyst (species.yr)	1,41E-11			9.07E-10			3.99E-11		
Terrestrial acidification (species.yr)	6,93E-11			3.10E-09			1.37E-10		
Freshwater eutrophication (species.yr)	1,14E-11			2.51E-09			1.06E-10		
Marine eutrophication (species.yr)	3,66E-14			4.51E-13			1.92E-14		
Terrestrial ecotoxicity (species.yr)	1,92E-12			4.89E-11			2.38E-12		
Freshwater ecotoxicity (species.yr)	2,17E-12			1.16E-10			5.20E-12		
Marine ecotoxicity (USD2013)	4,06E-13			2.34E-11			1.05E-12		
Land use (species.yr)	9,59E-11			1.24E-09			5.28E-11		
Water consumption Terr ecosyst (species.yr)	7,68E-11			1,04E-09			1,92E-10		
Water consumption. Aqua ecosyst (species.yr)	3,43E-15			4,64E-14			8,58E-15		
Mineral resource scarcity (USD2013)	9,44E-05	3,19E-03	3,19E-03	1,15E-03	3.34E-01	3.34E-01	7,32E-05	1,45E-02	1,45E-02
Fossil resource scarcity (USD2013)	3,09E-03			3,33E-01			1,44E-02		
Total monetary (USD)			1.73E-02			1.26E+00			5.74E-02

* CFC-11 = trichlorofluoromethane; NOx = nitrogen oxides; 1.4-DCB = 1.4-dichlorobenzene

† refer to the process flow diagram in Supplementary Figure 4a. All assumptions and data sources are summarized in Supplementary Table 3.

‡excluding dyestuff. dyestuff is accounted for under “Production”.

Supplementary Table 3: Assumptions and sources for TEA and LCA.

Streams	Orientation	Associated Unit Operation	Amount	TEA assumptions and sources	LCA assumptions and sources (Ecoinvent 3.6 process)
**Aniline**	Input	A1	0.931 kg	Indoxyl price was estimated based on indigo price. Nevertheless, a mass balance balanced block flow diagram was generated to account for environmental impact of the process.<br><br>Process conditions are stated in the Supplementary Figure 2A	Aniline {RoW}l market for aniline l Cut-off, U
**Hydrogen Cyanide**	Input	A1	0.268 kg		Hydrogen cyanide {RoW}l market for hydrogen cyanide l Cut-off, U
**Formaldehyde**	Input	A1	0.297 kg		Formaldehyde {RoW}l market for formaldehyde l Cut-off, U
**Water**	Input	A1	0.17 l		Emissions to water
**Wastewater**	Output	A1	0.075 l		Wastewater from textile production {GLO}l market for wastewater from textile production l Cut-off, U
**Sodium Hydroxide**	Input	A2	0.497 kg		Sodium hydroxide, without water, in 50% solution state {GLO}l market for l Cut-off, U
**Steam**	Input	A2	125 kg		Steam not included. Information not available in ecoinvent 3.6
**Phenylglycine nitrile**	Intermediate Input	A2			Not included. Information not available in ecoinvent 3.6
**Water**	Input	A2	0.17 l		Water, unspecified natural origin, very high water stress
**Ammonia**	Output	A2	0.151		Emission to environment. Ammonia emissions to air
**Wastewater**	Output	A2	0.225 l		Wastewater from textile production {GLO}l market for wastewater from textile production l Cut-off, U
**Ammonia**	Intermediate	A3	0.305 kg		Ammonia, liquid {RoW}l market for l Cut-off, U
**Sodium**	Input	A3	0.412 kg		Sodium {RoW}l chloride electrolysis l Cut-off, U
**Hydrogen**	Output	A3	0.017 kg		Emission to environment. Hydrogen emissions to air
**Wastewater**	Output	A3	0.007 l		Wastewater from textile production {GLO}l market for wastewater from textile production l Cut-off, U
**Phenylglycine sodium salt**	Intermediate	A4			Not included. Information not available in ecoinvent 3.6
**Sodium amide**	Intermediate	A4			Not included. Information not available in ecoinvent 3.6
**Ammonia**	Output	A4	0.287		Emission to environment. Ammonia emissions to air
**Sodium Hydroxide**	Output	A4	0.337		Emission to environment. Sodium Hydroxide emissions to water
**Wastewater**	Output	A4	0.297 l		Wastewater from textile production {GLO}l market for wastewater from textile production l Cut-off, U
**Indoxyl**	Input	A5		Process conditions are stated in the Supplementary Figure 2A<br><br>Conventional indigo dyeing has been simulated based on an average recipe for vat dyeing obtained from literature (https://doi.org/10.1016/B978-0-08-102043-2.00003-4, page 51-53)	Assessed based on TEA input and output assessment
**Wastewater**	Output	A3	0.144 l		Wastewater from textile production {GLO}l market for wastewater from textile production l Cut-off, U
**Indigo**	Input	A6			Assessed based on TEA input and output assessment
**Sodium hydroxide + Water**	Input	A6	3.004 kg		Sodium hydroxide, without water, in 50% solution state {GLO}l market for l Cut-off, U, Water, unspecified natural origin, very high water stress
**Dispersing agent + Wetting agent + Sodium hydrosulphite**	Input	A6	1.05 kg		Sodium hydrosulphite {GLO}l market for l Cut-off, U
**Dyeing effluent**	Output	A6	195.281 l		Wastewater from textile production {GLO}l market for wastewater from textile production l Cut-off, U
**Indigo dyed denim**	Output (product)	A6	246.31 m2 (97 adult jeans)		Basis for LCA functional unit

**Sodium Phosphate buffer**	Input	B5	1.61 kg	Production estimates follows lab-based experimental scheme	Sodium phosphate {RER}l market for sodium phosphate l Cut-off, U
**Indoxyl**	Intermediate	B5			Part of total LCA for Indoxyl and Indican
**BGL enzyme + Dyeing buffer**	Input	B6	0.045 kg		Enzymes {RER}l enzymes production l Cut-off, U
**Undyed denim fabric**	Input	B6		Production estimates follows lab-based experimental scheme.<br>Process conditions are stated in the Supplementary Figure 2A	Production of denim fabric not included in the LCA
**Indican**	Intermediate	B6			Basis for LCA functional unit
**Dyeing effluent**	Output	B6			Part of total LCA for Indoxyl and Indican
**Dyed denim**	Output (product)	B6	229.2 m2 (90 adult jeans)		Basis for LCA functional unit
**Water, deionised**	Input	B6	286 kg		Water, deionized {Europe without Switzerland}l market for water, deionised l Cut-off, U
**Wastewater**	Output	B6	286 kg		Wastewater from textile production {GLO}l market for wastewater from textile production l Cut-off, U
**Electricity**	Input	C6	0.38 kWh		Electricity, medium voltage {RER}l market group for l Cut-off, U
**Dyeing buffer**	Input	C6	10.93 kg		Water, deionised {RoW}l market for water, deionised l Cut-off, U
**Undyed denim fabric**	Input	C6			Production of denim fabric not included in the LCA
**Indican**	Intermediate	C6			Not included of LCA assessment due to lack of information
**Dyeing effluent**	Output	C6			Part of total LCA for Indoxyl and Indican
**Dyed denim**	Output (product)	C6	One pair of dyed adult jeans		Basis for LCA functional unit

{RoW} = rest of the world (excluding Europe) market average production
{RER} = European market average production
{GLO} = global market average production
Cut-off, U = allocation method

Supplementary Table 4: Primers used in this study.

Introduced mutation	Primer forward (sequence 5'-3')	Primer reverse (sequence 5'-3')
P14C	ATG CCA CGU CAT AAT CGT GCC CTC CGC CGG C	ACG TGG CAU GGT GGA GCG GTG GTT GGT
A117C	ACC TTT UCG CCA CTG ATG CAA TCG ACG T	AAA AGG UCG ACG ACG AGG GCG CAG ACG CGG CGG CCG GAG
S21C	ATC GTG CCC UGC GCC GGC ATG GGC CAC CTC AT	AGG GCA CGA UTA TGA CGT GCG GTG GTG G
D122C	ACT GAT GCA AUC GAC GTC GCC CTT GAG CTC	ATT GCA TCA GUG GCG AAA AGG CAG ACG ACG AGG GCG GCG
V76C / R97C	ACG CCC AAA UCG AGA CTC TCA TGT CCC TCA TGG TTG TCT GCT CCC TCC CCT CGC TCC GC	ATT TGG GCG UCG GAG GGG GCG TCG GAG AGG TCG CAC TCG GGG AGG AAG GAG GT
L119C / A132C	CC TTT UCG CCA CTG ATG CAA TCG ACG TCT GCC TTG AGC TCG GCA TCC GCC CTT T	AAA AGG UCG ACG ACG CAG GCG GCG ACG CGG CGG C
V121C / A125C	ACC TTT UCT GCA CTG ATG CAA TCG ACG TCG CCC	AAA AGG UCG CAG ACG AGG GCG GCG ACG CG
T126C	ATG CAA UCG ACG TCG CCC TTG AGC T	ATT GCA UCG CAG GCG AAA AGG TCG ACG AC
R208C	AAG TGC TAU AAA TTG GCC GAG GGT GTT ATC GTA	ATA GCA CTU GGA GTG GTG GAG GAG CCA CTT
A132C / I137C	AGC TCG GCU GCC GCC CTT TCA TCT TCT TCC CCT CC	AGC CGA GCU CAA GGC AGA CGT CGA TTG CAT CAG T
A132C / P139C	AGC TCG GCA UCC GCT GCT TCA TCT TCT TCC CCT CCA CCG CC	ATG CCG AGC UCA AGG CAG ACG TCG ATT GCA TCA GT
A147C	ACC TGC AUG ACC CTC TCC TTC TTC CT	ATG CAG GUG GAG GGG AAG AAG ATG AAA G
P227C	AGG GGG GAU GCA TCA GGG AGC TTT TGC ACC CC	ATC CCC CCU CCA AAC CCT CGA AGC TAT
P173C / P181C	ATC CCC GGG UGT ATT TGC GTC CAC GGC AAG GAT TTG ATC GAC	ACC CGG GGA UCT GAA CGC AGT CGG ACA GCT CGG CAA A
I176C	AGT GCC CCG GGU GTA TTC CGG TCC ACG GCA AGG ATT	ACC CGG GGC ACU GAA CCG GGT CGG ACA GCT
D186C	AGT GCT UGA TCG ACC CGG TTC AGG ATA GGA	AAG CAC UTG CCG TGG ACC GGA ATA C
K396C	AAT GCA UGA ACG CTG TTA TGC TAA CCG AGG G	ATG CAT UGC TCT GCA TAG AGG GGC CAT GT
P190C / A198C	AGA ACG ACU GCT ACA AGT GGC TCC TCC ACC ACT CC	AGT CGT TCU TCC TAT CCT GAA CGC AGT CGA TCA AAT CCT TGC C
D193C / N196C	AGG AAG UGC GAC GCC TAC AAG TGG CTC CTC C	ACT TCC UGC ACT GAA CCG GGT CGA TCA A
A259C / L264C	AGT GCT GCA AGU GGT TGG ACC AGC AGC CAC GTG GAT	ACT TGC AGC ACU CAG GCC GGC AAG CTG CCC CCT TCT CGC A
P271C / S274C	AGT GCC GUG GAT GCG TCC TAT TCG TGA ATT TCG GGA GT	ACG GCA CUG CTG GTC CAA CCA CTT CA
G273C	ACG TTG CUC CGT CCT ATT CGT GAA TT	AGC AAC GUG GCT GCT GGT CCA ACC ACT T
G365C	ACG TGC GGG UTC TTG ACG CAT TGT GGG TGG AAT T	ACC CGC ACG UCG ACT CAT GGC TTA AGA C
V278C	ATT CTG CAA UTT CGG GAG TGG TGG GGT C	ATT GCA GAA UAG GAC GGA TCC ACG TGG C
W307C	ATG CGT GGU TAG GCC TCC AAA CGA CGG CAT TG	ACC ACG CAU AGG AAC CTC TGC TGG CTG
L286C / Q290C	AGT ACG GAG UGC CAG AAC GAG CTT GCA GGT GTG C	ACT CCG TAC UGC AGA CCC CAC CAC TCC CGA AA
G337C / E340C	AGT GCT TCU TGT GCC AGA CCG CGG GCA GGG GTT T	AGA AGC ACU CGG GCA GGA GTT TCA ACG
T342C / G346C	AGG TGC UTG GTC TTG CCA ATG TGG GCC CC	AGC ACC UGC CCG CGC ACT GCT CCA AGA ACC CCT C
L368C / I387C	AGA GCG UGT TCC ATG GGG TAC CAC TAT GCA CAT GGC CCC TCT ATG CAG AGC AA	ACG CTC UCC AGT GTT GAA TTC CAC CCA CAA TGC GTG CAG AAC CCG CCC GTC GAC TCA
T388C / A399C	AGC AAA AGA UGA ACT GCG TTA TGC TAA CCG AGG GCC TGA GG	ATC TTT TGC UCT GCA TAG AGG GGC CAG CAA ATT AGT GGT ACC CCA TGG AAC A
P412C / E424C	ATG GAA UCA TCC GAG GTG CTT GCA TCG CAC GAG TTA TAG GGG AGT TG	ATT CCA UCC TTA CCC ACT GAG CAT CTG AGT CCC ACC CTC AG
V455C / S462C	AGC AAA GAU GGA TCA TGC ACT CGA GCT CTT GAA GAG GTT GCA	ATC TTT GCU CAA GCA AGC AGA AGC CGC ACG CTT
S457C / G460C	AAA GAT UGC TCA TCT ACT CGA GCT CTT GAA GAG	AAT CTT UGC ACA ATA CAG CAG AAG CCG C
L64C / I68C	ACA CCT CCU TCC TCC CCG AGG TCG ACC TCT	AGG AGG TGU CGC AGG AGG CAG GGC AGG AGG AGA GGA AGT CGC G
T146C/M148C	ACC CTC UCC TTC TTC CTC CAC CTC GAG AAG C	AGA GGG UGC AGG CGC AGG AGG GGA AGA AGA TGA AAG
A147C	ACC TGC AUG ACC CTC TCC TTC TTC CT	ATG CAG GUG GAG GGG AAG AAG ATG AAA G
E224C	AGG GTT TGU GCG GGG GAC CGA TCA GGG AGC TTT	ACA AAC CCU CGA AGC TAT TTA CGA TAA CA
E235C / K238C	ATG CCC GCG GGU TTA CCC GGT CGG ACC GCT GAT T	ACC CGC GGG CAU CCC GGG CAG GGG TGC AAA AGC TCC CTG AT
L267C	AG TGG UGC GAC CAG CAG CCA CGT GGA T	ACC ACT UCA AGC ACT CAG GCC GGG C
S363C	AGC CAT GAG UGC ACG GGC GGG TTC TTG ACG CAT T	ACT CAT GGC UTA AGA CAT CGA TCT GCG GGG
N279C	TT CGT GUG CTT CGG GAG TGG TGG GGT C	ACA CGA AUA GGA CGG ATC CAC GTG GC
V308C	ATG GTG CGU TAG GCC TCC AAA CGA CGG CAT TG	ACG CAC CAU AGG AAC CTC TGC TGG CTG
P390C/Q395C	ATG CAG AGU GCA AGA TGA ACG CTG TTA TGC TAA CC	ACT CTG CAU AGA GGC ACC ATG TAA TTA GTG GTA CCC
G460C/T463C	ATT GCT CAU CTT GCC GAG CTC TTG AAG AGG TTG CAA A	ATG AGC AAU CTT TGC TCA ATA CAG CAG AA
P48C	ACC TTC GCC GUA TGC ACC AGC GGC CCG CCC TCA	ACG GCG AAG GUG AAG GTG AAG CGC GGA AG
S98C	ATG GTT GUC CGC TGC CTC CCC TCG CTC CGC GAC CTC AT	CA ACC AUG AGG GAC ATG AGA GTC TCG ATT TG
P13C	ACC GCT CCA UGC CCG CAC GTC ATA ATC GTG	ATG GAG CGG UGG TTG GTG GAG CGG CGG G
A66C	ATC GAC ACC UCC TTC CTC CCC GAG GTC GAC C	AGG TGT CGA UGG AGC AAG GGA GGG AGG AGA GGA A
T10C	ACC TGC GCU CCA CCA CCG CAC GTC ATA	AGC GCA GGU TGG TGG AGC GGC GGG GGA
S112C	ACT CCG CCU GCG GCC GCC GCG TCG CCG CC	AGG CGG AGU AGG AGG CAA TGA GGT CGC
A22P	ATC GTG CCC UCC CCG GGC ATG GGC CAC CTC ATC	AGG GCA CGA UTA TGA CGT GCG GTG GTG G
M91I	ATC TCC CUC ATG GTT GTC CGC TCC CTC CCC	AGG GAG AUG AGA GTC TCG ATT TGG GCG
E75P	ACC TCC TUC CTC CCC CCG GTC GAC CTC TCC GAC GCC CC	AAG GAG GUG TCG ATG GAG GCA GGG AG
E157P	ACC TCC CGA AGC UTG ATG AAA CGG TGT CAT GTG AGT T	AGC TTC GGG AGG UGG AGG AAG AAG GAG AGG GTC AT
G222D	AGG ATT UGG AGG GGG GAC CGA TCA GGG	AAA TCC UCG AAG CTA TTT ACG ATA ACA
G405D	AGG ACC UGA GGG TGG GAC TCA GAC CCT CAG T	AGG TCC UCG GTT AGC ATA ACA GCG TTC A
G409A	AGG GTG GCA CUC AGA CCC TCA GTG GGT AAG GAT GG	AGT GCC ACC CUC AGG CCC TCG GTT AGC AT
G430K	ATA AAA GAG UTG ATG GAA GGT GAG GAA GGG AAA C	ACT CTT TTA UAA CTC GTG CGA TCT CAG C
G222D (45)	AGG ATT UGG AGG GGG GAC CGA TCA G	AAA TCC UCG AAG CTA TTT ACG ATA ACA
G405D / G409A / G430K	AGG ATG GAA UCA TCC GAG GTG CTG AGA TCG CAC GAG TTA TAA AAG AGT TGA TGG AAG GTG AGG AAG GG	ATT CCA TCC UTA CCC ACT GAG GGT CTG AGT GCC ACC CTC AGG TCC TCG GTT AGC ATA ACA GCG
E75P (46)	ACC TCC TUC CTC CCC CCG GTC GAC CTC TCC GAC GCC	AAG GAG GUG TCG ATG GAG GCA GGG AG
E157P (46)	ACC TCC CGA AGC UTG ATG AAA CGG TGT CAT G	AGC TTC GGG AGG UGG AGG AAG AAG GAG AGG GTC A
G222D (46)	AGG ATT UGG AGG GGG GAC CGA TCA G	AAA TCC UCG AAG CTA TTT ACG ATA AC
S50D	ACG GCC CGC CCU CAT CCT CCC AGC GCG ACT T	AGG GCG GGC CGU CGG TGG GTA CGG CGA AGG T
M94T	ACG GTT GUC CGC TCC CTC CCC TCG CT	ACA ACC GUG AGG GAC ATG AGA GTC TCG ATT T
Q86K	ACG CCA AAA UCG AGA CTC TCA TGT CCC TCA TGG T	ATT TTG GCG UCG GAG GGG GCG TCG GAG A
Q86R	ACG CCC GUA TCG AGA CTC TCA TGT CCC TCA TG	ACG GGC GUC GGA GGG GGC GTC GGA GA
A107K	ATT AAA UCC TAC TCC GCC TCC GGC CG	ATT TAA UGA GGT CGC GGA GCG AGG G
K210R	ATC GTT UGG CCG AGG GTG TTA TCG TAA ATA G	AAA CGA UAC CTC TTG GAG TGG TGG A
Q269E	AGC CAC GUG GAT CCG TCC TAT TCG TGA ATT TC	ACG TGG CUG CTC GTC CAA CCA CTT CAA GCA
V285T	ACC CTC AGU ACG GAG CAG CAG AAC GAG CTT	ACT GAG GGU CCC ACC ACT CCC GAA ATT
A299E	AGG TGT GCU GGA ACA CAG CCA GCA GAG GTT C	AGC ACA CCU GCA AGC TCG TTC TGC TGC
Q341R	AGG GGT TCU TGG AGC GTA CCG CGG GCA GGG GTT TGG	AGA ACC CCU CGG GCA GGA GTT TCA ACG
K332D / E336K / Q341R / A343K	AGG GTT CUT GGA GCG TAC CAA AGG CAG GGG TTT GGT CTT GCC AAT G	AGA ACC CUT TGG GCA GGA GAT CCA ACG GGT CGA TCT CCC C
S413K	ACC CAA AGU GGG TAA GGA TGG AAT CAT CCG AGG	ACT TTG GGU CTG AGT CCC ACC CTC AGG
I472K	AAA AAA UGG GAA AGC AAG GTT TAA GGA TCC T	ATT TTT UTG CAA CCT CTT CAA GAG C
A81L	ACC TGC CCU CCG ACG CCC AAA TCG AGA CTC T	AGG GCA GGU CGG AGA GGT CGA CCT CGG
S110V	CG TGG CCU CCG GCC GCC GCG TCG CCG	AGG CCA CGU AGG AGG CAA TGA GGT CGC
I129F	ATT CGA CGU CGC CCT TGA GCT CGG CAT C	ACG TCG AAU GCA TCA GTG GCG AAA AGG
I188L	ATT TGC UGG ACC CGG TTC AGG ATA GGA AGA AC	AGC AAA UCC TTG CCG TGG ACC GGA AT
L333F	AAT TCC UGC CCG AGG GGT TCT TGG AGC	AGG AAT UTC AAC GGG TCG ATC TCC C
M351S	AGG GGT TUG GTC TTG CCA AGC TGG GCC CCG CAG ATC GAT GT	AAA CCC CUG CCC GCG GTC TGC TCC AA
F381V	AGC GTG GUC CAT GGG GTA CCA CTA ATT ACA TGG	ACC ACG CUC TCC AGT GTT GAA TTC CAC
T388A	ATT GCA UGG CCC CTC TAT GCA GAG CAA AAG	ATG CAA UTA GTG GTA CCC CAT GGA A
S55A	AGC GCG ACU TCC TCT CCT CCC TCC CTG CCT	AGT CGC GCU GGG AGG CTG AGG GCG GGC CGC TGG T
A125G	ACC TTT UCG GCA CTG ATG CAA TCG ACG TCG CC	AAA AGG UCG ACG ACG AGG GCG GCG A
F140Y	ACA TCT UCT TCC CCT CCA CCG CCA T	AAG ATG UAA GGG CGG ATG CCG AGC TC
I472K	AAA AAA UGG GAA AGC AAG GTT TAA GGA TCC T	ATT TTT UTG CAA CCT CTT CAA GAG CT
GV296/297LG	ACT GGG UCT GGC CCA CAG CCA GCA GAG GTT	ACC CAG UGC AAG CTC GTT CTG CTG C
H300M	AGG TGT GCU GGC CAT GAG CCA GCA GAG GTT CCT ATG GG	AGC ACA CCU GCA AGC TCG TTC TGC TGC
E340G	AGG GGT TCU TGG GCC AGA CCG CGG GCA GGG GTT T	AGA ACC CCU CGG GCA GGA GTT TCA ACG
F381V (In Mut 87 context)	AGC GTG GUC CAT GGG GTA CCA CTA ATT GCA TGG	ACC ACG CUC TCC AGT GTT GAA TTC CAC
A388C/A399C (In Mut 88 context)	AGC AAA AGA UGA ACT GCG TTA TGC TAA CCG AGG GCC TGA GG	ATC TTT TGC UCT GCA TAG AGG GGC CAG CAA ATT AGT GGT ACC CCA TGG

FROM INDIGO TO INDICAN

Elaborating Social Sustainability Potentials in Relationship to a more Environmentally Sustainable Process of Indigo Dyeing with Indican

ABSTRACT

This report discusses some of the potential social effects related to introducing an alternative, more eco-friendly dyeing process that innovates denim dying by replacing indigo with its precursor indican. Indican is soluble and stable, and both indican production as well as dyeing can be achieved by employing enzymatic processes, which alleviating the environmental footprint of denim dyeing considerably.

Based on two local case studies that where selected due to the volume of denim yarn they produce, located in dyeing hubs in India and China, the report explores how communities in close proximity to dyeing facilities are affected by conventional indigo dyeing. As it is estimated that 20% of the global industrial water pollution is caused by textile dyeing[3], the focus of this report rest primarily with water pollution and its effects on communities. The discussions are framed through the lens of social sustainability, focusing especially on the principle of *providing a good quality of life,* in regards to socio-economic as well as health related issues.

The aim of the report is to provide a first, provisional framework to better understand and contextualize the potential advantages of indigo dyeing with indican. By framing the discussions through the lens of social sustainability, the report seeks to highlight some of the aspects that should be considered when implementing indigo dyeing with indican, which are related to yet go beyond environmental advantages.

CONTENT

1. INTRODUCTION 3

1.1 From Indigo to Indican 3
1.2 Framing Social Sustainability 4
1.3 Research Question and Scope of this Report 5

2. INTRODUCING INDICAN - ASPECTS OF SOCIAL SUSTAINABILITY 7

2.1 Environmental and Social Consequences of Textile Dyeing 7
2.2 Water Pollution and its Effects on Local Communities 7
2.2.1 Textile Dyeing in Pali City 8
2.2.2 Textile Dyeing in Xintang, China 9
2.3 Discussion 9
2.3.1 Scenario 1: Indigo Dyeing with Indican is Introduced in India and China 10
2.3.2 Scenario 2: Indigo Dyeing with Indican is Introduced in Europe 10
2.3.3 Localism vs. Environmental Sourcing 10

3. END-USER ATTITUDES TOWARDS SUSTAINABLE GARMENTS 12

3.1 Discussion 14

4. CONCLUDING REMARKS 14

SUPPLEMENTARY REFERENCES 16

1. Introduction

The textile and apparel industry with its complex and globally operating supply chains has in recent years been increasingly criticized for its devastating environmental impact as well as its adverse effects on human life quality, especially within production countries[4]. The problems are complex, wide-reaching and interrelated in diverse ways, ranging from environmental issues relating to *e.g*. the use of pesticides that decrease biodiversity or the pollution of water through waste chemicals, and social ones such as child labour in the cotton industry and dangerous and unhealthy working conditions in production. Moreover, high amounts of overproduced garments are eventually destroyed as they cannot be sold and fast fashion items that do not last longer than one season are over-consumed and end too quickly in landfills. The problems hence span throughout the entire life-cycle of garments, thereby connecting the sourcing of raw materials, the production as well as consumption and disposal of clothes in ways that seem inherently unsustainable given the high environmental and social costs.

Efforts towards a more sustainable textile and apparel industry therefore have to be thought of and implemented in ways that address and account for this complexity of processes and relationships. Particularly, technological innovations should be understood and implemented to not only advance the environmental impacts of garments throughout their life-cycle (*e.g.* resource reduction, chemical pollution) but also advance social and cultural sustainability. Otherwise, they might end up as "techno-fixes (that) target issues in isolation"[5], not accounting for the interdependency of issues and concerns, fixing one problem while creating new ones.

Sustainability in the textile and apparel industry has thus to be addressed in more systemic ways, that pay attention to the interrelatedness of the system in its whole. Consequently, this report aims at providing a provisional framework that situates the implementation of an environmentally more sustainable indigo dye into a broader context of potentially changing relationships, explored through the lens of social sustainability.

1.1 From Indigo to Indican

Latest estimations valued the global denim jeans market at approximately USD 63.5 billion in 2020 with an expectation to rise towards USD 87.4 billion by 2027, at a CAGR of around 4.7%[6]. With the overall heightened focus on increasing sustainability in the textile and apparel industry, the denim industry is also moving towards more sustainable production solutions.

It is estimated that the production of a pair of jeans consumes >10,000 L of water (cotton growing, dyeing and processing of denim), about 500 g of chemicals (dyes, auxiliaries and finishing agents) and a vast amount of energy (irrigation of cotton, denim processes such as spinning, weaving, processing and sewing)[7]. These quantities need to be multiplied by two billions to arrive at the consumption of the world's total jeans production for a year. Hence, the question of more sustainable production arises. This report focuses on the stage of denim dyeing and the potentiality of increased social sustainability by introducing an environmentally more sustainable dyeing process.

Indigo is generally regarded as the oldest known natural dye throughout the world, with the oldest piece of dyed cloths dating back 6000 years[8]. Throughout its history, the colorant has been retrieved from organic materials, mostly plants from the *Indigofera* family.

With an increasing demand for indigo dye brought forward by advancing technological opportunities for the mass production of textiles, natural indigo dye couldn't fulfil the new market's demands satisfactory. After years of experimentation, in 1880 Adolf von Baeyer produced the first synthetic indigo dye, which eventually led to the industrial synthesis of indigo by the BASF AG in 1897[9]. With the introduction and commercialization of synthetic indigo, the market for natural indigo dye rapidly lost in share.

Synthetic indigo paved the way for the mass production of denim yarn and was one of the key factors for the success of the iconic garment known as blue jeans. However, the denim industry is causing tremendous environmental and social hazards in its production countries, and textile dyeing is often regarded as one of the most polluting parts during the production phase. The iconic blue denim dye, indigo, is especially problematic due to its insolubility and large environmental footprint. It has proven hard to substitute with other blue pigments since the particular hue and ring colouring property of indigo cannot be obtained with any other known blue pigment.

The insoluble nature of indigo necessitates the use of harsh chemicals in the dyeing process. This has led to different innovations that tried to tackle the environmental problems caused by dyeing denim yarn and several approaches have been tested and/or implemented. One such effort is the use of biological indigo, which has been proposed but was never fully implemented, probably due to not being economically sustainable on the large scale[10]. Right now, pre-reduced indigo, *i.e.* synthetic indigo to which reducing chemicals are already added, is the state of the art of alternative indigo dyeing. In this form, the use of alkali and additional reducing agents during the dyeing process can be reduced, yet not fully replaced.

To alleviate this, it has been suggested to use the natural indigo precursor indican instead. Indeed, indican is soluble and stable, and both indican production and indigo dyeing with indican can be achieved employing enzymatic processes. Indican therefore can be said to advance the environmental sustainability of denim dyeing by substituting the use of harsh chemicals with processes happening under mild conditions. From an environmental perspective, dyeing with indican thus would reduce the amount of hazardous chemicals that are being used during yarn dyeing, and their concentrations in effluents.

1.2 Framing Social Sustainability

The report's aim is to emphasize social aspects related to the implementation of indican dye into the current industrial system. To do so, the report applies a social sustainability framework to discuss the cases presented here. Even though complementing economic and environmental sustainability in the three-pillar model of sustainable development, social sustainability remains the pillar least studied[11]. This might be due to the lack of a common definition of which factors to include and address and the difficulties of applying comparable as well as quantifiable categorizations in relationship to social aspects[12,13]. Yet, this level of sustainability can help in detecting and highlighting stakeholders, relationships, interests, problems and consequences that often go under the radar of solely economically and environmentally framed assessments of sustainability.

According to the Western Australian Council of Social Service (WACOSS)

> Social sustainability occurs when the formal and informal processes, systems, structures, and relationships actively support the capacity of current and future generations to create healthy and liveable communities. Socially sustainable communities are equitable, diverse, connected, and democratic and provide a good quality of life[14].

Drawing on this broad understanding, in this report questions of social sustainability are framed by the last overreaching principle, being *providing a good quality of life.* Here, it is foremost socio-economic as well as health concerns relating to denim dyeing and how they might get affected by the implementation of indigo dyeing with indican that will be discussed. Outlining the current state based on two cases, this report focuses on the pollution of waterways and its health related as well as socio-economical consequences for local communities. The aim thus is to provide a first, provisional framework to better understand and contextualize the potential advantages following an introduction of indigo dyeing with indican through the lens of social sustainability.

1.3 Research Question and Scope of this Report

The overreaching research question that guides the exploration of this report is the following:

What might be some of the social effects following the implementation of indican into the current system?

To explore this rather broad question, the report is divided into two parts that emphasize two different entry points into the current system, referring broadly to the side of production and consumption. In part 1 the focus will be on the implementation of indigo dyeing with indican in the production side of the denim industry. Here, to provide a more in-depth understanding of the potential changes, the report is based on two hypothetical scenarios:

1. **Scenario 1: Dyeing with Indican is introduced in India and China**
2. **Scenario 2: Dyeing with Indican is introduced in Europe**

These countries/regions are chosen based on their current position within the market, with India and China producing the biggest volume of dyed denim yarn. Europe here functions as a potential go-to market for the newly developed enzymatic dyeing process.

The report will briefly outline the current state within the production countries in relationship to the involved industries and how they affect surrounding, local communities, framed through two specific, regional cases. With a focus on water pollution caused by the textile dyeing industry, the report seeks to identify how the implementation of indigo dyeing with indican might improve the livelihood of communities in close proximity to dyeing mills, while also highlighting the potentialities of moving part of the production to Europe.

The second part of the report highlights end-consumer perspectives on buying more sustainable garments, exploring the European market potential for more sustainably dyed denim and end-consumer attitudes towards sustainable denim products.

Rather than producing definite claims about the social sustainability of indigo dyeing with indican, the reports seeks to provide a provisional framework that highlights some of the contextual relationships being potentially affected by its implementation. It thus aims at highlighting some of the social aspects that often get overlooked when sustainability only gets framed through environmental advantages.

It should also be noted that the report only uses secondary resources in the discussion and therefore has to rely on the data available. However, the textile and apparel industry is notorious for its secrecy and lack of transparency in regards to suppliers and subcontractors, making it rather difficult to obtain reliable data and information on the conditions of workers within the industry in general. It seems further difficult to obtain data that specifically discusses the conditions in, and impact of, denim dyeing facilities. Therefore, the report is built on more general data on the textile industry and dyeing processes.

PART 1

2. Introducing Indican - Aspects of Social Sustainability

The denim industry operates across different continents and countries, and includes various processes, from harvesting the cotton raw material to selling finished products in retail. It is further regarded to be one of the most fragmented sub-industry within the textile and apparel market[15]. This report focuses on one part of the system, namely the dyeing process of denim yarn where the introduction of indigo dyeing with indican has the potential to make a positive environmental impact.

The current Covid-19 pandemic has once again made visible the tremendous inequality that is built into the system of the textile and apparel industry, especially in the production countries, causing mass unemployment, and leaving workers with no pay for already executed work. Increasing food insecurity in the end of the supply chain of the textile industry is one of the harsh realities of the working conditions that have become even harsher under the current pandemic[16]. A general lack of job security the further one moves down the line within supply chains, paired with often unsafe and unhealthy working conditions, makes the industry not only one of the most polluting ones but also a particularly problematic one in regards to the conditions it provides for the work force it employs. Yet, the industry is also estimated to employ 75 million people globally, 85% of them being women, creating opportunities to move from informal to formal work[17].

In general, the direct environmental impacts caused by the textile industry are highly localized. Foremost those communities that are in close proximity to industrial areas have to deal with the consequences of e.g. toxicity caused through production. In terms of the broader effects, the communities surrounding textile producing facilities “are significantly impacted in terms of health, quality of life and, in many cases, by associated impacts on their livelihoods from farming and fishing”[17]. This is due to the increasing pollution of waterways through wastewaters released by the textile industry. In the following, the current state of affairs in two countries, namely India and China, will be briefly outlined. The report focuses on regional examples based on case study reports.

2.1 Environmental and Social Consequences of Textile Dyeing

Dyeing is commonly regarded as one of the most polluting stages within textile production due to the use of harsh chemicals in textile dyes[18]. Besides the potentially harmful consequences related to direct exposure to chemicals during textile dyeing, the process is especially damaging for surrounding communities when it comes to water. On the one hand, the high amounts of water used within the dyeing phase can cause serious problems for regions where access to clear drinking water is already under pressure. On the other hand, it is estimated that 20% of global industrial water pollution is caused by the textile dyeing and finishing industry[3], entering local waterways, polluting the water supply for local communities. In the following, the report discusses some of the severe consequences that affect local communities in close proximity to textile dyeing mills when effluents are discharged in waterways without proper treatment.

2.2 Water Pollution and its Effects on Local Communities

The two cases this report will present are Pali city, India and Xintang, China. Where Pali city is a general dyeing hub, with many different dyeing processes taking place, Xintang is known as “denim capital of the world” and estimated to produce 60% of China’s denim.

2.2.1 Textile Dyeing in Pali City

Being the administrative headquarter of Pali district, Rajasthan, Pali city holds one of the biggest textile dyeing and bleaching hubs in India, with around 800 operating textile mills. Situated on the banks of Bandi River, the city was identified as one of the critically polluted areas by the Central Pollution Control Board in 1998[19] and has been a case example for the hazardous pollution of the textile dyeing industry and the influences this has on the livelihood of communities in near proximity. The case also highlights some of the policy as well as governmental problems such as a lack of sufficient control and actions taken that might prevent further pollution.

The problems in the area started to intensify with increasing market demand for textile production, which resulted in ever more textile dyeing mills opening up. A case study research conducted by the Centre for Science and Environment (CSE), an Indian public interest research and advocacy organisation, found that 80% of the water samples they collected in five different areas where “not fit to be used under any class of surface water”[20].

Further, the serious pollution of the groundwater decreased the quality of soil for agricultural uses, causing a reduction in income as well as food supply for farmers in the surrounding communities, forcing many of them into wage labour[21]. Thus, the pollution of Bandi river by the textile dyeing industry has had tremendous effects on the socio-economic conditions of the nearby population, due to loss of clean water sources and agricultural land mass.

For nearly three decades farmers from 50 nearby villages have organised themselves to protest the pollution caused by the dyeing mills, which not only affects the river and the groundwater in the area, causing the agricultural fields to become infertile, but also the water in the Nehra dam 40 km downstream, which was initially built to collect rainwater[21].

Besides the scientific proof of the pollution of waterways, anecdotal evidence shows incidents of fish dying and livestock being affected by being exposed to polluted water sources. Yet, no health survey was ever conducted, hampering evaluation and anticipation of the extent of negative effects of the textile dyeing industry on the health of its surrounding communities[22]. Another problem highlighted by activists as well as the CSE is that even though several court decision prevent the textile mills in Pali to release untreated wastewater into the river, there seems to be no real legal punishment for those mills not complying and no sufficient governmental action to improve the situation in any significant way.

The case of Pali city is a good example for the hazardous, localized consequences of severe pollution by textile mills. The pollution of waterways as well as groundwater, leading to infertility of agricultural soil, changes not only the socio-economic structures of communities[19], but is also a health hazard through consumption of polluted water either directly or indirectly.

2.2.2 Textile Dyeing in Xintang, China

Commonly referred to as "the denim capital of the world", Xiantang is located in the northeastern part of the Pearl River Delta. Chinese authorities estimate that the region produces around 300 million denim articles per year, employing around 220.00 people; many of them migrating from other parts of China.

Although an economically successful region, Xintang suffers from severe pollution due to the high concentration of especially textile mills in the area. A report released by Greenpeace in 2009[23], investigating the pollution of waterways by the local industry, indicates that many factories, including textile dyeing mills, dumb their wastewater into the river without sufficient treatment, causing the water to turn black-blue. Based on water samples taken from five different pipes, the report demonstrates that the water released contained excessive amounts of heavy metals, organic pollutants and chemicals.

Ever since China opened up its economy to the global market in the late 1970s, the country has held a strong position in the production of all kinds of consumer goods and textiles have historically been an important part of this economic success. Nonetheless, the Chinese environment as well as population have paid and are still paying a high price for this rapid industrialisation: By now, 70% of Chinas rivers and other water sources are polluted[24], some of them to such a high degree that the water cannot (or rather should not) be consumed under any circumstances. Xintang is just one of many examples for the severe consequences of industrial pollution. Local communities suffer heavily from the pollution of waterways as they depend on these nearby water sources in their daily lives. By now, the pollution of the Pearl River is so severe that it might threaten the water supply of bigger cities further downstream.

To tackle the severe problems caused by the textile industry in the area, the local government in Xintang established the new Xinzhou Environmental Industry Park and moved about 80 dyeing mills into the area. Yet, rather than solving the pollution problem, it only got replaced to a new location and smaller textile dyeing mills continue to dump their untreated wastewater into the river. One of the problems pointed out by environmental activist is that the sheer number of industrial facilities makes it nearly impossible to comprehensively control them, creating loopholes for non-compliance.

Even though there is no scientific evidence for increased health problems in the communities in close proximity to Xintang, there are many anecdotal testimonies from community members blaming the denim industry for health problems ranging from skin rashes to infertility.

2.3 Discussion

Taking its point of departure from the two case presented, the following discussion will elaborate on some of the issues that should be considered when assessing the implementation of indigo dyeing with indican from a social sustainability perspective.

The state of the art treatment of wastewaters and effluents released from textile dyeing mills is currently centred on "end-of-pipe measures" such as wastewater treatment plants. Yet, as research suggests, these plants are not efficient enough to fully treat the effluents and many organic pollutants "will either pass through the treatment process unchanged, be converted through partial degradation into other hazardous substances, or accumulate in treatment plant 'sludges' that then become hazardous wastes in themselves"[23]. Therefore

it is suggested that “the most effective measures to address hazardous substances are those that seek alternatives to the use of such hazardous substances in manufacturing processes, progressively replacing them with less hazardous (…) alternatives in order to bring about rapid reductions”[23]. It is in this regard that indigo dyeing with indican could arguably make an impact on the wastewater that is released from dyeing mills, positively impacting the water quality of communities in close proximity to textile dying facilities.

2.3.1 Scenario 1: Indigo Dyeing with Indican is Introduced in India and China

As the two cases from India and China demonstrate, the consequences of water pollution by textile dyeing facilities are severe for surrounding communities. Given the outlined current situation, it can be anticipated that a decrease of toxins that are released during the processes of denim yarn dyeing through the implementation of indigo dyeing with indicant will lead to lesser pollution of wastewater. This would have positive outcomes for the surrounding communities affected by the pollution. Yet, it is difficult to determine how much of a difference the implementation of indican would actually make as there is no reliable data available that specifically looks at the pollution caused by solely indigo dyeing. Indeed, many dyeing hubs do not exclusively specialize in denim yarn dyeing, even though indigo is the most used garment dye. Nonetheless, every step towards more environmentally sustainable dyeing processes is a step towards decreasing the severe socio-economic and health related consequences that communities in close proximity to textile dyeing industries are facing.

2.3.2 Scenario 2: Indigo Dyeing with Indican is Introduced in Europe

In the European context, “water is one of the most comprehensively regulated areas of EU environmental legislation”[25], providing a strong legal framework for the treatment of wastewaters from e.g. the textile dyeing industry. If the implementation of indigo dyeing with indican is moved to dyeing mills in Europe it could therefore be argued that local communities in close proximity to denim dyeing mills are already well protected against the harmful consequences following water pollution. In fact, two of the most well-known and biggest denim hubs in Europe are located in Italy and Spain, and are often praised for their sustainable and innovative manufacturing processes. Both facilities have a long-standing, locally embedded and internationally recognized tradition in denim manufacturing, highlighting craftsmanship, tradition and social and environmental consciousness. Strategically, it could therefore be argued that these facilities might have an increased interest in advancing their environmentally conscious production by implementing indigo dyeing with indican into their operation and they could very well function as case study examples for the implementation.

2.3.3 Localism vs. Environmental Sourcing

The idea of localism has lately entered debates on fashion sustainability and has been praised for a potential future trajectory in creating more sustainable business operations. “Localism is defined as a business strategy where companies try to establish a supply chain in geographic proximity, while consciously taking into account local conditions in the business decisions”[26]. The introduction of the process of indigo dyeing with indican into the European context could be thought of as a move towards more localism in the denim industry by creating supply chains that are closer to their main target market. This move towards more localism in the denim industry, moving part of the production of denim yarn to regions where the finished products also will be sold, could open up for a sustainability potential that relates to heightened transparency on production level as the legal frameworks in Europe are quite regulative.

Moving a bigger part of the denim supply chain to Europe by implementing indigo dying with indican into European dyeing mills can also help in establishing more green jobs. In the Nordic region, the Council of Nordic Ministers has *e.g.* published a report, which investigates the regions potential for a continuing transition into a greener textile and apparel industry, highlighting the ambition that sees it as an opportunity for green growth[27].

Yet, it should also be kept in mind that if the more environmentally friendly dyeing process of indican is moved to dyeing mills in Europe, the largest market share of denim dyeing would still rest in countries like India and China, thereby contributing to the continued outsourcing of environmentally harmful industrial processes while implementing greener solutions in areas where legal framework and systems of control are already better implemented. This consideration relates to claims made by a number of authors that see environmental sourcing as a major driver when determining where to locate aspects of the supply chain that have high levels of environmental impact[28–30]. In this argumentation, "environmental sourcing refers to multi-national corporations (MNCs) strategically locating operations in countries that are still developing their environmental regulatory systems, such as Bangladesh, China, India, Indonesia and Viet Nam, in order to exploit regulatory uncertainty"[17].

Another aspect that might be relevant to consider in this context is job security for workers in current production countries. Would an introduction of indigo dyeing with indican in the European market move a significant amount of jobs from current production countries to Europe? As indican probably would not be implemented on a large scale immediately, it is rather difficult to anticipate what the consequences in regards to job security in current production countries would be. Yet, these questions are relevant. From a social sustainability perspective, it should therefore be considered where the biggest harm for people and their livelihood is done currently and where the biggest advances could be achieved, in regards to environmental, health-related as well as socio-economic impacts. The most important question to ask seems therefore: Who should benefit from the more environmentally sustainable dyeing process?

PART 2

3. End-User Attitudes towards Sustainable Garments

In this second part of the report, the market for more sustainably dyed denim products will briefly be investigated. The data used in this report is drawn from Fashion Revolutions latest Consumer Survey Report released in December 2020[31]. Fashion Revolution is a non-for-profit organization, advocating for systemic change within the fashion industry by mobilising citizens, industry and policymakers through research, education and other advocacy work. The survey was conducted as part of an end line study in the 'Trade Fair, Live Fair' project 2017-2020, funded by the European Commission. Around 5000 respondents in the age group 16-75 answered the survey, coming from the five largest European markets, Germany, France, Italy, Spain and the UK. Even though the data does not specifically relate to denim products as such, its broader insights on consumer attitudes in relationship to the environmental as well as social impacts of garments and the chemical exposure caused by them is a useful starting point to explore the potential for more sustainably dyed denim products in the European market.

Denim products can generally be said to have a high sustainability potential in the use-phase, given the fabrics durability and therefore potential for extended usage. As a fabric, denim is resilient and long lasting, and even though the colour in denim garments is fading through continuous washing, garments tend to age in attractive and desirable ways. These attributes make denim a well-suited fabric to investigate sustainability potentials in the use phase of garments.

As was highlighted in the first part of this report, textile dyeing can cause tremendous environmental and socio-economic harm, when wastewater is not sufficiently treated. With a heightened awareness in the general public of the severe environmental as well as social consequences of the textile and apparel industry, in this second part of the report an overview is given that situates the potential for denim products dyed in a more sustainable way into broader discussions on Fashion Sustainability.

In the following, key findings from the Consumer Survey Report will be presented. Those findings are not ordered in the same way as in the initial report, rather the focus is on the data relevant within the scope of this report*.

* A break down of the percentages per country is provided in the initial report

People’s attitudes on environmental impacts of garments

78% of people agreed that it’s important for brands to share detailed information about their products environmental impacts.

76% of people agreed that fashion brands should be required by law to protect the environment at every stage of making and selling their products.

75% of people agreed that fashion brands should be required by law to provide information about the environmental impacts of their business.

33% of people said it’s important that the clothing (including shoes & accessories) they buy is produced in a way that is not harmful to the environment.

21% of people have tried to purchase clothing that is made in an environmentally sustainable way.

18% of people have tried not to purchase clothing where they have concerns about the environmental impact.

People’s attitudes on social impacts of garments

79% of people agreed that fashion brands should be required by law to respect the human rights of everybody involved in making their products.

75% of people agreed that fashion brands should do more to improve the lives of the women making their clothes, shoes or accessories.

72% of people agreed that fashion brands should be required by law to provide information about the social impacts of their business.

22% of people said it is important that the clothing they buy is produced in safe working conditions.

19% of people have tried to purchase clothing that is made in a socially responsible way.

16% of people have tried not to purchase clothing where they have concerns about the items’ impact on society.

Other relevant issues

14% of people said it is important that the clothing they buy is produced locally.

37% of people said it is important that the clothing they buy is produced without using harmful chemicals for the consumer.

69% of people would like to know how their clothes were manufactured.

69% of people are interested in learning about what fashion brands do to reduce their waste.

3.1 Discussion

These numbers suggest that there is a good strategic fundament in regards to implementing the more environmentally sustainable process of indigo dyeing with indican in general. Indeed, around 75% of consumers in the five biggest European markets utter a general concern about the environmental as well as social impacts of the garments they purchase. Further, a high amount of consumers also thinks that there should be legal frameworks in place that hold brands accountable for the social as well as environmental impacts of their businesses. To implement such accountability, a heightened transparency about the production processes is necessary and here indigo dyeing with indican could make a good case, shedding light on an often unaddressed issue in discussions of Fashion Sustainability, namely textile production and the social and environmental impacts of this part of the supply chain. The EU does regulate the amount of chemicals that are allowed in textiles and garments that are sold within its markets, and many brands have taken steps to guarantee that none of the chemicals classified as severely toxic can be found in textiles and garments sold to end-users. Yet, there has not been any comprehensive focus on the use and release of chemicals during the production phase; and with stricter regulations in the global North and higher costs connected to wastewater treatment, many brands have moved their polluting production stages to countries in the global South. The process of dyeing indigo with indican therefore tackles an issue that is often overlooked in Fashion Sustainability, where there has been much focus on garment workers, neglecting those workers and local communities even further down the supply chain.

4. Concluding Remarks

Considering that environmentally sustainable innovations should be thought of and implemented in relationship to the wider systems they will become part of, this report discussed some of the potential social effects related to introducing indigo dyeing with indican into the current system. Based on two case studies on major production sites in India and China, the report explored what the consequences of the textile dyeing industries are right now, in relationship the living conditions of surrounding communities. The discussions were framed through the lens of social sustainability, focusing especially on the principle of *providing a good quality of life,* in regards to health related issues and job security. The report focuses on water pollution caused by textile dyeing (responsible for 20% of the worldwide water pollution) and the severe consequences of this pollution for communities in close proximity to dyeing facilities. Drawing on these two cases, the report presented some of the problems faced by local communities. The discussion then centred on two hypothetical scenarios for the implementation of the more environmentally sustainable process of indigo dyeing with indican, the first being an implementation in regions that currently hold the biggest market share of denim yarn dyeing (India and China) and a potential go-to market in Europe.

Summing up, indigo dyeing with indican has the potential to make a difference for communities in close proximity to textile dyeing facilities, especially in regions where legal frameworks are weak when it comes to holding textile producers accountable for the pollution they cause. From a social sustainability perspective, implementing indigo dyeing with indican in countries like India and China would potentially make the biggest positive impact for the people directly impacted by the pollution caused by textile dyeing mills.

1. Lack of data and transparency

To make more substantial claims about the social sustainability of indigo dyeing with indican, more research is needed on the subject matter. There is a general problem, in regards to comprehensive and reliable data when it comes to supply chains in the textile and apparel industry[32]. To make more well-assessed statements about the impacts on social sustainability it is therefore important to conduct more research into the actual working conditions of people employed in the denim dyeing process as well as case studies analysing the actual effects, specifically of denim yarn dyeing on communities surrounding dyeing mills. Unfortunately, there is not much research done on health related issues working with chemicals in the textile dyeing phase and much evidence on health related issues comes from individual and anecdotal testimonies.

2. Best for whom?

From a social sustainability perspective, the overall question one should ask oneself in regard to the implementation of green solutions more generally speaking and specifically for the implementation of indigo dyeing with indican is: where could the biggest impact be achieved in relationship to peoples well-being (individual and community level)?

3. Good timing for more sustainable dyeing processes

There is a heightened focus on more sustainable production processes in the textile and apparel industry. The ongoing EU Textile Initiative is a good example of the importance and the momentum that more sustainable textile production and consumption currently hold. Therefore, indigo dyeing with indican could and should be implemented in accord to these initiatives.

Supplementary References

1. Bungaruang, L., Gutmann, A. & Nidetzky, B. Leloir Glycosyltransferases and Natural Product Glycosylation: Biocatalytic Synthesis of the C -Glucoside Nothofagin, a Major Antioxidant of Redbush Herbal Tea. *Adv Synth Catal* **355**, 2757–2763 (2013).
2. Ögmundarson, Ó., Sukumara, S., Herrgård, M. J. & Fantke, P. Combining Environmental and Economic Performance for Bioprocess Optimization. *Trends Biotechnol* **38**, 1203–1214 (2020).
3. Kant, R. Textile dyeing industry an environmental hazard. *Nat Sci (Irvine)* **04**, 22–26 (2012).
4. Fletcher, K. & Tham, M. *Earth Logic : Fashion Action Research Plan*. (JJ Charitable Trust, 2019). https://earthlogic.info/wp-content/uploads/2021/03/Earth-Logic-E-version.pdf
5. Ceschin, F. & Gaziulusoy, I. Evolution of design for sustainability: From product design to design for system innovations and transitions. *Des Stud* **47**, 118–163 (2016).
6. Global Industry Analysts 2021. Denim Jeans. Global Market Trajectories and Analytics. Preprint at https://www.strategyr.com/market-report-denim-jeans-forecasts-global-industry-analysts-inc.asp (2021).
7. Sustainable denim manufacturing process creates 'green' jeans. *The American Chemical Society* (2012). https://www.acs.org/content/acs/en/pressroom/newsreleases/2012/june/sustainable-denim-manufacturing-process-creates-green-jeans.html
8. Li, S., Cunningham, A. B., Fan, R. & Wang, Y. Identity blues: the ethnobotany of the indigo dyeing by Landian Yao (Iu Mien) in Yunnan, Southwest China. *J Ethnobiol Ethnomed* **15**, 13 (2019).

9. Schmidt, H. Indigo – 100 Jahre industrielle Synthese. *Chemie in unserer Zeit* **31**, 121–128 (1997).

10. Berry, A., Dodge, T., Pepsin, M. & Weyler, W. Application of metabolic engineering to improve both the production and use of biotech indigo. *J Ind Microbiol Biotechnol* **28**, 127–133 (2002).

11. Murphy, K. The social pillar of sustainable development: a literature review and framework for policy analysis. *Sustainability: Science, Practice and Policy* **8**, 15–29 (2012).

12. Littig, B. & Griessler, E. Social sustainability: a catchword between political pragmatism and social theory. *International Journal of Sustainable Development* **8**, 65 (2005).

13. Boström, M. A missing pillar? Challenges in theorizing and practicing social sustainability: introduction to the special issue. *Sustainability: Science, Practice and Policy* **8**, 3–14 (2012).

14. Nilipour, A. (Azi). Introduction to Social Sustainability. in *Social Sustainability in the Global Wine Industry* 1–14 (Springer International Publishing, 2020). doi:10.1007/978-3-030-30413-3_1.

15. Annapoorani, S. G. Introduction to denim. in *Sustainability in Denim* 1–26 (Elsevier, 2017). doi:10.1016/B978-0-08-102043-2.00001-0.

16. Kyritsis, P., LeBaron, G. & Nova, S. *Hunger in the Apparel Supply Chain: Survey findings on workers' access to nutrition during Covid-19*. https://www.workersrights.org/wp-content/uploads/2020/11/Hunger-in-the-Apparel-Supply-Chain.pdf (2020).

17. *Green Jobs and a Just Transition for Climate Action in Asia and the Pacific*. https://www.ilo.org/wcmsp5/groups/public/---asia/---ro-bangkok/---sro-bangkok/documents/publication/wcms_734887.pdf (2019).

18. Mia, R. *et al.* Review on various types of pollution problem in textile dyeing & printing industries of Bangladesh and recommandation for mitigation. *Journal of Textile Engineering & Fashion Technology* **5**, (2019).

19. Bhadra, B. K., Pathak, S. & Sharma, J. R. Impact of industrial effluents on groundwater around Pali city, Rajasthan using field and satellite data. *Journal of the Geological Society of India* **82**, 675–691 (2013).

20. Tripathi, P., Pranaw, K., Sahu, R. & Saxena, P. *Evaluation of Water Pollution due to Textile Industries in Pali, Rajasthan*. (2014) doi:10.13140/RG.2.1.1358.6086.

21. Bhaduri, A. For a better Bandi. *India Water Portal* (2017). https://www.indiawaterportal.org/articles/better-bandi

22. Tur, J. K. Environment panel to visit pollution-hit Pali villages. *The Times of India* (2016). https://timesofindia.indiatimes.com/spotlight/learn-the-latest-in-data-science-and-machine-learning-to-grow-your-career/articleshow/104855019.cms?utm_source=contentofinterest&utm_medium=text&utm_campaign=cppst

23. Brigden, K., Labunska, I., Santillo, D. & Johnston, P. *Hazardous Chemical Pollution of the Pearl River. Investigation of chemicals discharged with wastewaters from five industrial facilities in China*. https://greenpeace.to/publications/hazardous-chemical-pollution-o.pdf (2009).

24. *Poisoning the Pearl. An investigation into industrial water pollution in the Pearl River Delta*. https://issuu.com/greenpeace_eastasia/docs/poisoning-the-pearl (2010).

25. Cattoor, T. European legislation relating to textile dyeing. in *Environmental Aspects of Textile Dyeing* (ed. Christie, R. M.) 1–29 (Elsevier, 2007). doi:10.1533/9781845693091.1.

26. Dybdahl, L. M. Business Model Innovation for Sustainability Through Localism. in 193–211 (2019). doi:10.1007/978-3-319-97385-2_11.

27. Klepp, I. G. *et al. Mapping sustainable textile initiatives*. (Nordic Council of Ministers, 2015). doi:10.6027/TN2015-545.

28. Anguelov, N. *The Dirty Side of the Garment Industry Fast Fashion and Its Negative Impact on Environment and Society*. (CRC Press, Taylor & Francis Group, 2016).

29. Khan, M. S., Ahmed, S., Evans, A. E. V. & Chadwick, M. Methodology for Performance Analysis of Textile Effluent Treatment Plants in Bangladesh. *Chemical Engineering Research Bulletin* **13**, (2009).

30. Greer, L., Egan Keane, S. & Lin, Z. *NRDC's Ten Best Practices for Textile Mills to Save Money and Reduce Pollution*. https://www.nrdc.org/sites/default/files/rsifullguide.pdf (2010).

31. *Consumer Survey Report. Fashion Revolution 2020.* https://issuu.com/fashionrevolution/docs/fashrev_consumersurvey_2020_full (2020).

32. Skjold, E. Modebranchens Datakrise. *Weekendavisen* (2021). https://www.weekendavisen.dk/2021-51/ideer/modebranchens-datakrise?fbclid=IwAR0l6zu9u76my4eP1QYEeUFylQQ7DvJwcMlCEhyP3pTRVSHtqUv6U5tsHoo
